# Glioblastoma stem cells express non‐canonical proteins and exclusive mesenchymal‐like or non‐mesenchymal‐like protein signatures

**DOI:** 10.1002/1878-0261.13355

**Published:** 2023-01-16

**Authors:** Haris Babačić, Silvia Galardi, Husen M. Umer, Mats Hellström, Lene Uhrbom, Nagaprathyusha Maturi, Deborah Cardinali, Serena Pellegatta, Alessandro Michienzi, Gianluca Trevisi, Annunziato Mangiola, Janne Lehtiö, Silvia Anna Ciafrè, Maria Pernemalm

**Affiliations:** ^1^ Department of Oncology and Pathology Karolinska Institute, Science for Life Laboratory Stockholm Sweden; ^2^ Department of Biomedicine and Prevention University of Rome Tor Vergata Italy; ^3^ Department of Immunology, Genetics and Pathology Uppsala University Sweden; ^4^ Unit of Immunotherapy of Brain Tumors, Department of Molecular Neuro‐Oncology, Foundation IRCCS Institute for Neurology Carlo Besta Milan Italy; ^5^ Neurosurgical Unit Hospital Spirito Santo, Pescara, “G. D'Annunzio” University Chieti Italy

**Keywords:** glioblastoma stem cells, mesenchymal, proneural, proteogenomics, proteomics, signature

## Abstract

Glioblastoma (GBM) cancer stem cells (GSCs) contribute to GBM's origin, recurrence, and resistance to treatment. However, the understanding of how mRNA expression patterns of GBM subtypes are reflected at global proteome level in GSCs is limited. To characterize protein expression in GSCs, we performed in‐depth proteogenomic analysis of patient‐derived GSCs by RNA‐sequencing and mass‐spectrometry. We quantified > 10 000 proteins in two independent GSC panels and propose a GSC‐associated proteomic signature characterizing two distinct phenotypic conditions; one defined by proteins upregulated in proneural and classical GSCs (GPC‐like), and another by proteins upregulated in mesenchymal GSCs (GM‐like). The GM‐like protein set in GBM tissue was associated with necrosis, recurrence, and worse overall survival. Through proteogenomics, we discovered 252 non‐canonical peptides in the GSCs, i.e., protein sequences that are variant or derive from genome regions previously considered non‐protein‐coding, including variants of the heterogeneous ribonucleoproteins implicated in RNA splicing. In summary, GSCs express two protein sets that have an inverse association with clinical outcomes in GBM. The discovery of non‐canonical protein sequences questions existing gene models and pinpoints new protein targets for research in GBM.

## Graphical abstract

Cancer stem cells (GSCs) drive malignancy in glioblastoma. However, their molecular phenotype is not well understood. Here, we report proteomic profiling of GSCs and protein sets that separate two GSC types, which are differentially associated with overall survival in glioblastoma. Through proteogenomics, we detect matching mRNA and protein sequences mapping to gene regions previously considered non‐coding for proteins, including variants of HNRNPs.

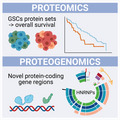

**Abbreviations**
CGP: chemical and genetic perturbations
CPTAC: Clinical Proteomic Tumor Analysis Consortium
CTmvp: cellular tumor, microvascular proliferation
CTpan: cellular tumor, palisading around necrosis
EGFR: epidermal growth factor receptor
EMT: epithelial‐to‐mesenchymal transition
FDR: false discovery rate
GBM: glioblastoma
GM‐like: GSAPS mesenchymal‐like protein set
GPC‐like: GSAPS proneural and classical‐like protein set
GSAPS: GSC‐associated proteomic signature
GSCs: glioblastoma cancer stem cells
GSEA: gene set enrichment analysis
H: hallmark
HGCC: human glioma cell culture
HiRIEF LC–MS/MS: high‐resolution isoelectric focusing coupled with liquid chromatography and mass‐spectrometry
HNRNPs: heterogeneous ribonucleoproteins
IEF: isoelectric focusing
IPG: immobilized pH gradient
KM: Kaplan–Meier curve
LE: leading edge of the tumor
lncRNAs: long non‐coding RNAs
MET: mesenchymal epithelial transition gene
MSigDb: Molecular Signatures Database
OS: overall survival
PCA: principal component analysis
PMT: proneural‐to‐mesenchymal transition
PSMs: peptide spectrum matches
RNAseq: RNA sequencing
RRID: Research Resource Identifiers
ssGSEA: single‐sample gene set enrichment analysis
TMT: tandem mass tags
UMAP: Uniform Manifold Approximation and Projection

## 1 Introduction

Glioblastoma (GBM) is the most common malignant primary brain tumor, almost inevitably fatal, and characterized by short survival [1, 2, 3]. A previous GBM molecular classification proposed by Verhaak et al. [4], based on mRNA expression patterns, distinguished four GBM subtypes: classical, mesenchymal, proneural, and neural. More recently, the classification was revised by removing the neural subtype and highlighting subtypes' plasticity, i.e. the ability to switch from one subtype to another [5]. The gene expression in adult GBM tumors has been further explored by the Clinical Proteomic Tumor Analysis Consortium (CPTAC) at several levels, including the proteome, which led to a new, multiomic classification of GBM tumor subtypes to: nmf1 (proneural‐like), nmf2 (mesenchymal‐like), and nmf3 (classical‐like) [6].

Extensive research about the origin of GBM has established the theory that cancer stem cells drive the development and progression of GBM, contribute to resistance to chemo‐ and radio‐therapy, and induce GBM recurrence [7, 8]. Primary GBM stem cells (GSCs) have shown to reflect the diversity of GBM, recapitulate the tumor subtypes at mRNA level, and represent a good model to study the molecular profile of this cancer and explore new therapeutic targets [9]. Many efforts were undertaken to uncover gene expression signatures that are pivotal for GSC functions, expanding our understanding of the transcriptome and proteome of GBM and GSCs [9, 10, 11, 12, 13, 14, 15, 16, 17, 18]. Single‐cell RNA‐sequencing studies have demonstrated that GSCs are plastic and can switch between different subtypes [19]. Despite these efforts to characterize the transcriptional programs responsible for GSCs' plasticity and stemness, no study has provided in‐depth proteomic or proteogenomic profiling of primary GBM stem cells. Furthermore, it is not known how well GBM subtypes are recapitulated in GSCs at protein level.

The aim of this study was to explore the proteomic and proteogenomic landscape of GSCs, to enhance our comprehension on: (a) protein signatures that would define GSC characteristics; (b) the relation between mRNA and protein levels in GSCs; (c) how the expression of GSC signatures relates to disease aggressiveness in GBM cancer tissue; and (d) the existence of non‐canonical peptides originating from genome regions previously considered as non‐protein‐coding.

**Fig. 1:**
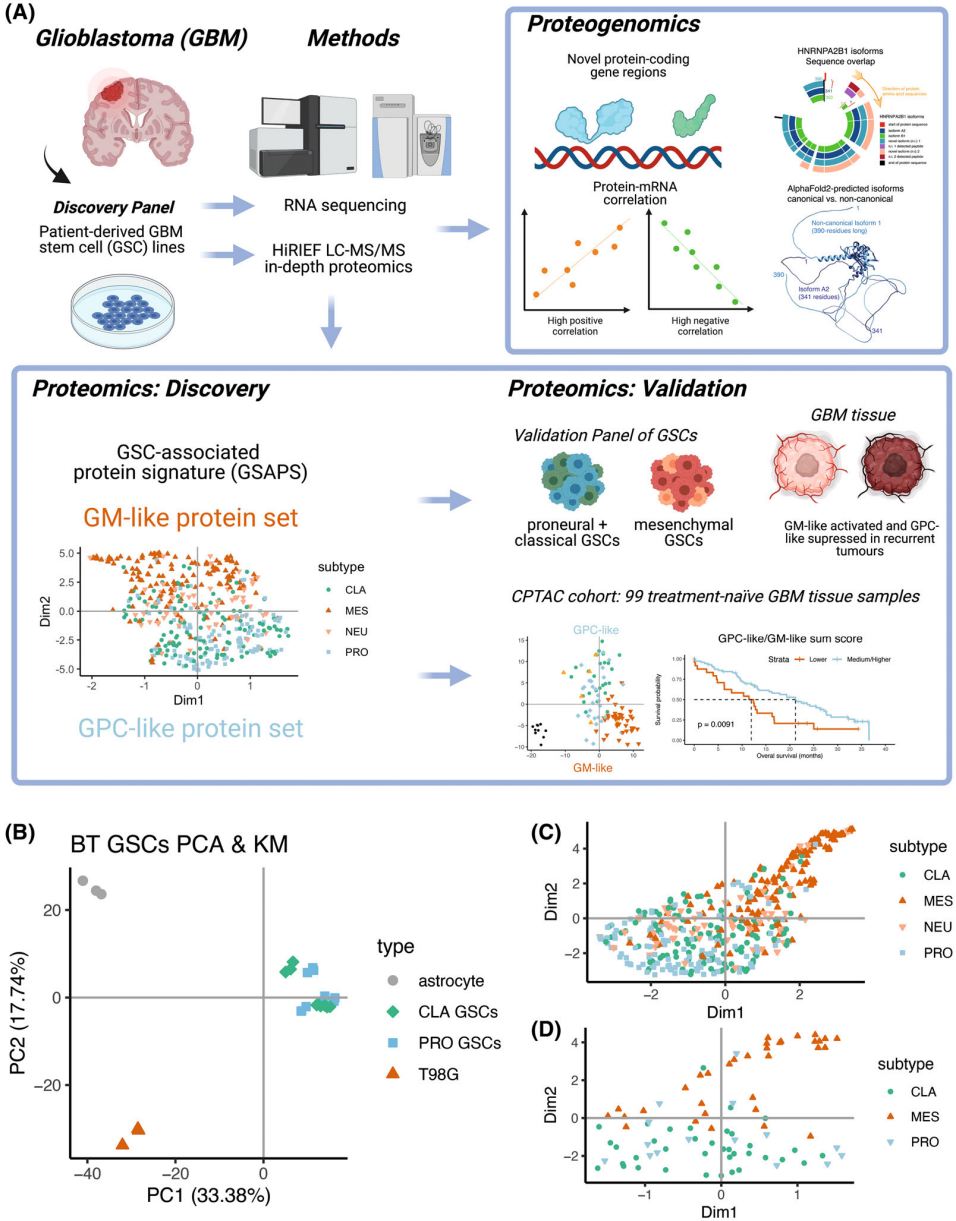
Study workflow and exploratory findings. (A) In a discovery panel of six patient‐derived GSC lines, previously subtyped as expressing the classical and proneural GBM subtype at mRNA level, we have identified variable enrichment of the proneural (PRO) and classical (CLA) GBM subtype, suggesting a plasticity between the two subtypes. However, all the GSC lines had a suppression for the GBM mesenchymal (MES) subtype at protein level. We hypothesized that the GSCs are more distinctive at protein level based on whether they express the mesenchymal subtype or not and aimed to identify a protein signature (GSAPS), that consisted of two protein sets: The proneural and classical‐like (GPC‐like) protein set that was upregulated in proneural and classical GSCs and a mesenchymal‐like protein set (GM‐like) upregulated in mesenchymal GSCs. GSAPS was identifiable in another panel of 11 patient‐derived GSCs, and in GBM tissue, where the expression of higher GPC‐like protein scores was associated with better overall survival, whereas higher GM‐like protein scores were associated with worse overall survival. Finally, by integrating proteomic and transcriptomic expression, we have performed proteogenomic analysis of the discovery panel of GSCs, discovering novel protein‐coding gene regions and providing assessment of how well mRNA levels predict protein levels (figure created with BioRender.com); (B) PCA based on proteomic expression of the GSC samples and non‐GSC cell lines; (C, D) UMAP of protein products of genes included in the Verhaak 2010 GBM subtypes' gene sets (C) and Wang 2017 GBM subtypes' gene sets (D).

Here, we report deep transcriptome and proteome profiling of patient‐derived GSCs, by RNA‐sequencing (RNAseq) and high‐resolution isoelectric focusing coupled with liquid chromatography and mass‐spectrometry (HiRIEF LC–MS/MS), respectively. We discovered a GSC‐associated protein signature (GSAPS), which reflects two protein programs in GSCs that are inversely associated with clinical outcomes in GBM, one associated with the non‐mesenchymal (proneural and classical) subtypes and another associated with the mesenchymal subtype. We demonstrate that GSAPS recapitulates key features of GSCs and is associated with recurrence and overall survival (OS) in GBM patients. Furthermore, we report mRNA‐protein correlations and non‐canonical protein sequences in GSCs, discovering potentially new protein‐coding targets for research and treatment (Fig. 1A).

## 2 Materials and methods

### 2.1 GSCs and GBM tumors

#### 2.1.1 Ethical considerations

All patients gave written informed consent to use the samples for experiments, and the study methodologies were in line with the standards outlined in the Declaration of Helsinki. Processing of the BT human GSC lines and HGCC GSC lines was approved by the institutional Ethical Committee of Fondazione IRCCS Istituto Neurologico C. Besta [20] and the Uppsala Ethical Review Board (ID: 2007/353), respectively. The experiments including the GBM tissue samples were approved by the Ethics Committee of the Provinces of Chieti and Pescara, and of the “G. d'Annunzio” University of Chieti‐Pescara (ID: 21052020).

#### 2.1.2 GSC cell lines

BT human GSC lines were derived from surgical samples of consecutive primary GBMs, obtained at the Neurological Institute of the Carlo Besta Foundation IRCCS according to a protocol approved by the institutional Ethical Committee, which was previously described and characterized [20, 21]. Briefly, BT GSCs were derived by mechanical dissociation and digestion of tumor specimens with collagenase type I (Life Technologies‐Invitrogen, Monza, Italy). Single‐cell suspensions were plated at clonal density (50 cells·μL^−1^) in standard medium containing: DMEM/F‐12 (1 : 1) (1×) + GlutaMAX (Gibco, Waltham, MA, USA) containing 1% penicillin/streptomycin (Corning), 1% l‐glutamine (Aurogene, Rome, Italy), 2% B27 (Gibco), 0.1% heparin (Sigma‐Aldrich, Waltham, MA, USA), 0.002% bFGF (PeproTech, Suzhou, China), and 0.002% EGF (PeproTech), at 37 °C in a humidified 5% CO_2_ incubator. The BT GSC stemness was characterized by assessing the expression of stem‐cell markers such as SOX2, NES, or CD133, and by performing *in vitro* clonogenic assays. The multipotency of BT GSCs was assessed by culturing dissociated neurospheres in pro‐differentiating conditions (single‐cell suspensions plated in pro‐adhesive flasks in DMEM/F‐12 containing 2 mm glutamine, penicillin–streptomycin, B‐27 and 10% fetal bovine serum), while measuring the expression of neural (MAP2), astroglial (GFAP), or oligodendroglial (GalC) markers. The *in vivo* tumorigenic potential of BT GSCs was previously characterized by subcutaneous injection into immunocompromised mice [20, 21]. For all experiments, GSCs were grown *in vitro* for < 10 passages.

The human glioblastoma T98G (RRID: CVCL_0556) cell line and the astrocyte line were purchased from ATCC and CliniSciences (Guidonia Montecelio, Italy), respectively. The T98G cells were cultured in DMEM supplemented with 10% fetal bovine serum. The astrocytes were cultured in Astrocyte Medium (ScienCell #1801, Carlsbad, CA, USA). We cultured three biological replicates for each BT GSC and the T98G line, and one biological replicate for the astrocyte line. All cell lines were authenticated by short tandem repeat (STR) profiling using GenePrint^®^ 10 System by Promega Italia (Milano, Italy) in the past 3 years before the experiments. All cell lines were mycoplasma free (analyzed by PCR) at the time of experiments.

The human glioma cell culture (HGCC) GSC lines (RRIDs: CVCL_IR90, CVCL_IR82, CVCL_IS03) are part of the HGCC biobank (https://hgcc.se/) and have been previously described and validated [9, 22]. The cells were cultured as previously described, characterized for maintaining stemness potential [9, 22, 23], and analyzed between passages 10–19. Briefly, the cultures were maintained on poly‐ornithine/laminin‐coated dishes in DMEM/F12 Glutamax (Gibco) and Neurobasal medium (Gibco) mixed 1 : 1, with addition of 1% B27 (Invitrogen), 0.5% N_2_ (Invitrogen), 1% Penicillin/Streptomycin (Sigma), and 10 ng·mL^−1^ of each EGF and FGF2 (PeproTech). All HGCC cell lines were authenticated by STR profiling in the past 3 years before the experiments. They all have been regularly screened for mycoplasma infection using a PCR‐based method with the primers Myco1 (5′‐GGCGAATGGGTGAGTAACACG) and Myco2 (5′‐CGGATAACGCTTGCGACTATG) (Invitrogen) and no cultures have tested positive.

#### 2.1.3 GBM tumor tissue processing

Ten GBM tissue samples were collected and freshly frozen at the Neurosurgical Unit and evaluated by a pathologist at the Hospital Spirito Santo, Pescara, “G. D'Annunzio” University, Chieti, Italy. The tissue samples were fixated on an OCT compound and cut into 10 μm‐thick sections with a kryotome, of which 30 sections were collected in a tube for lysis and parallel sections were fixed on slides for hematoxylin and eosin staining. The sections collected in tubes were washed in PBS to remove the blood, centrifuged, and the tissue pellets were used for subsequent DNA, RNA, and protein isolation with the AllPrep DNA/RNA/Protein Mini Kit (Qiagen, Hilden, Germany).

### 2.2 RNA sequencing

Sequencing libraries for whole transcriptome analysis were prepared using Stranded mRNA‐Seq Library Preparation Kit. RNAseq was performed on an Illumina HiSeq 2500 Sequencer using standard conditions at the Next Generation Sequence Facility of University of Trento (CIBIO, Trento, Italy).

#### 2.2.1 RNA isolation, library preparation, RNA‐sequencing, qRT–PCR

Total RNA from the BT GSCs and the non‐GSC cell lines was isolated by TRIzol (Invitrogen), subjected to DNase‐I (Ambion, Thermo Fisher Scientific, Waltham, MA, USA) treatment, and RNAs were depleted of ribosomal RNA. Two RNA samples derived from normal brain were purchased from Clontech Laboratories (Takara Bio Europe, Saint‐Germain‐en‐Laye, France) and BioChain (Newark, CA, USA), respectively, and underwent RNAseq.

#### 2.2.2 Data quality check

The fastq files generated by the Illumina sequencer were monitored for quality by the FastQC tool (https://www.bioinformatics.babraham.ac.uk/projects/fastqc/, v.0.11.6). It provides a modular set of analyses that tests if the data has any problems. Since for each sample there is one FastQC output, with several results, we used the MultiQC tool (http://multiqc.info/, v.1.4) to aggregate the information for a better interpretation. The main outcome of these analysis is that the reads have very good quality and despite some differences among samples, the further analyses were performed without corrections at this stage.

#### 2.2.3 Transcript quantification

Transcript quantification was performed using Salmon [24]. Salmon applies a quasi‐mapping with a two‐phase inference procedure to quantify expression at the transcript level. The unique feature that distinguishes Salmon from other transcript assemblers is in its ability to account for experimental and other biases that are common to RNAseq data, such as GC content. Ensembl cDNA release 99 from GRCh38 was used as the target transcriptome. To obtain gene‐level quantifications, the median value across the transcripts of each gene was assigned as the gene expression. All options were set to default and −l A parameter was set to detect the library type from the RNAseq datasets.

### 2.3 Mass‐spectrometry‐based proteomics

The samples were prepared and analyzed following the HiRIEF LC–MS/MS protocol, as previously described [25]. Brief description is provided below.

#### 2.3.1 Cell lysis and in‐solution digestion

The BT GSCs were lysed in 200 μL SDS‐lysis buffer (containing 4% (w/v) SDS, 50 mm HEPES pH 7.6, and 1 mm dithiothreitol) using 1 : 4–10 of sample to buffer ratio. Afterwards, the cells were heated at 95 °C for 5 min while shaking on a pre‐warmed block and sonicated to dissolve the pellet and disrupt the remaining DNA. The lysate was then centrifugated at 14 000 ***g*** for 15 min and the supernatant removed. Proteins from HGCC cells and GBM tissue were extracted with the AllPrep DNA/RNA/Protein Mini Kit (Qiagen).

The protein concentration in the lysate was determined by Bio‐Rad DC Assay (Hercules, CA, USA) and equal amounts of each sample was subjected to in‐solution digestion. Briefly, the cell pellet was denatured at 95 °C for 5 min followed by reduction with dithiothreitol and alkylation with chloroacetamide at end concentrations of 5 and 10 mm, respectively. Lys‐C was added at a 1 : 50 (w/w) ratio and digestion was performed at 37 °C, 6 h or overnight. The samples were further digested by trypsin at a 1 : 50 (w/w) ratio with 37 °C overnight incubation. After LysC/trypsin digestion, ~ 1% of each peptide sample was aliquoted for ~ 15 min gradient LC–MS/MS runs to check for protease activity by the samples' miscleavage rate.

#### 2.3.2 TMT‐labelling

Before labelling, equal amounts of peptide samples were pH‐adjusted using TEAB, pH 8.5. The resulting peptide mixtures were labeled with Thermo Fisher Scientific isobaric Tandem Mass Tags (TMT). Biological triplicates of the BT GSCs and the T98G line, and technical triplicates of the astrocyte line were labeled with three TMT‐10‐plex sets, using two internal standards per set. The internal standards were made of sample pools. HGCC GSC samples were run in one TMTpro‐16‐plex set, without an internal standard, leaving the 133C and 134N channels empty. GBM tissue samples were labeled with one TMT‐10‐plex set, without an internal standard. Labelling efficiency was determined by LC–MS/MS before pooling of samples. Subsequently, sample clean‐up was performed by solid phase extraction (SPE strata‐X‐C; Phenomenex, Torrance, CA, USA). The labelling schemes per sets can be found in Tables S1–S3.

#### 2.3.3 High resolution isoelectric focusing (HiRIEF)

After sample clean‐up, the sample pool was subjected to peptide IEF‐IPG (isoelectric focusing by immobilized pH gradient) in pI range 3–10. The freeze‐dried peptide sample was dissolved in 250 μL rehydration solution containing 8 m urea and allowed to adsorb to the gel strip by swelling overnight. The 24 cm linear gradient IPG (Immobilized PH Gradient) strip (GE Healthcare, Chicago, IL, USA) was incubated overnight in 8 m rehydration solution containing 1% IPG pharmalyte pH 3–10 (GE Healthcare). After focusing, the peptides were passively eluted into 72 contiguous fractions with MilliQ water/35% acetonitrile/35% acetonitrile and 0.1% formic acid, using an in‐house constructed IPG extractor robotics (GE Healthcare Biosciences AB, Uppsala, Sweden; prototype instrument) into a 96‐well plate (V‐bottom, product #651201; Greiner Bio‐One, Kremsmünster, Austria). The BT GSCs samples were rerun and additionally fractionated by IEF‐IPG in pI range 3.7–4.9, to detect more peptides for proteogenomic analyses. The resulting fractions were then dried, frozen, and kept at −20 °C until LC–MS/MS analysis.

#### 2.3.4 LC–MS/MS analysis

Online LC–MS/MS was performed using a Dionex UltiMate™ 3000 RSLCnano System coupled to a Q‐Exactive HF mass spectrometer (Thermo Fisher Scientific). Each plate well was dissolved in 20 μL solvent A and 10 μL were injected. Samples were trapped on a C18 guard‐desalting column (Acclaim PepMap 100, 75 μm × 2 cm, nanoViper, C18, 5 μm, 100 Å), and separated on a 50 cm long C18 column (Easy spray PepMap RSLC, C18, 2 μm, 100 Å, 75 μm × 50 cm). The nano capillary solvent A was 95% water, 5% DMSO, 0.1% formic acid; and solvent B was 5% water, 5% DMSO, 95% acetonitrile, 0.1% formic acid. At a constant flow of 0.25 μL·min^−1^, the curved gradient went from 2% B up to 40% B in each fraction, followed by a steep increase to 100% B in 5 min and subsequent re‐equilibration with 2% B.

FTMS master scans with 60 000 resolution (and mass range 300–1700 *m/z*) were followed by data‐dependent MS/MS (30 000 resolution) on the top 5 ions using higher energy collision dissociation (HCD) at 30% normalized collision energy. Precursors were isolated with a 2 *m/z* window. Automatic gain control (AGC) targets were 1^6^ for MS1 and 1^5^ for MS2, with minimum AGC target of 1^3^. Maximum injection times were 100 ms for MS1 and 100 ms for MS2. The entire duty cycle lasted ~ 2.5 s. Dynamic exclusion was used with 30.0 s duration. Precursors with unassigned charge state or charge state 1, 7, 8, or > 8 were excluded.

#### 2.3.5 Protein identification

Raw MS/MS files were converted to mzML format using msconvert from the ProteoWizard tool suite [26]. Spectra were then searched in the Galaxy framework using tools from the Galaxy‐P project [27, 28], including msgf+ [29] (v.2020.03.14) and Percolator [30] (v.3.04.0), where eight subsequent HiRIEF search result fractions were grouped for Percolator target/decoy analysis. Peptide and PSM (Peptide Spectrum Matches) FDR (False discovery rate) were recalculated after merging the Percolator groups of eight search results into one result per TMT set. The reference database used was the human protein subset of Ensembl (v.101). Quantification of isobaric reporter ions was done using OpenMS project's IsobaricAnalyzer [31] (v.2.5.0). Quantification on reporter ions in MS2 was for both protein and peptide level quantification based on median of PSM ratios, limited to PSMs mapping only to one protein and with an FDR *q*‐value < 0.01. FDR for protein level identities was calculated using the −log10 of best‐peptide *q*‐value as a score. The search settings included enzymatic cleavage of proteins to peptides using trypsin limited to fully tryptic peptides. Carbamidomethylation of cysteine was specified as a fixed modification. The minimum peptide length was specified to be six amino acids. Variable modification was oxidation of methionine.

### 2.4 Proteogenomic identification

The proteogenomic pipeline is described in detail elsewhere [32]; a brief description is provided as follows. Transcripts were assembled from the RNAseq data of each sample using StringTie (v.2.113) [33] based on the human reference gene annotations (Ensembl, v.99). Next, transcripts with low expression level (TPM < 1) were removed and a peptide database was generated from the transcript sequences using custom scripts. Tryptic peptides with a minimum length of eight amino acids and a maximum length of 40 amino acids were kept. The database was fractionated based on the peptide isoelectric points as further detailed in [25]. Finally, the human canonical proteins (Ensembl, v.99) were appended to the peptide database.

The proteomics data from each cohort were searched against the peptide database from the same cohort using msgf+ Release (version 15 January 2020). Percolator (v.3.04.0) was used for Percolator target‐decoy scoring. Peptides at FDR < 1% were considered significant, while those matching canonical protein sequences were removed. Using blast, the remaining peptides were searched against a larger collection of reference protein databases that included UniProt (v.11, December 2019), gencode (v.33), Ensembl (v.99), and RefSeq (version 29 May 2020). Peptides matching any sequence were removed and those with one mismatch were further validated using SpectrumAI [34]. Finally, the list of novel peptides contained peptides with more than one mismatch or no match to known proteins as well as those that passed SpectrumAI.

### 2.5 Bioinformatics and statistical analyses

#### 2.5.1 Differential expression analysis and GSAPS derivation

Protein or peptide differential expression analysis was performed with a two‐sided *t* test for all comparisons and corrected for multiple testing with the FDR at 5%. The GSAPS was derived by comparing each BT GSCs triplicate to an astrocyte and T98G line triplicate and finding the intersect of proteins consistently upregulated and downregulated in the proneural and classical BT GSCs as compared to non‐GSC lines (Fig. S5). Those proteins that were upregulated in proneural and classical BT GSCs we named as the GPC‐like protein set, and those that were downregulated in BT GSCs that included many mesenchymal proteins we named as the GM‐like protein set.

We further refined the GSAPS by filtering the initial proteins that were included in the GPC‐like protein set to include only those proteins that were also upregulated in the proneural and classical GSCs from the HGCC panel, and the proteins included in the GM‐like set to include only those proteins that were also upregulated in the mesenchymal HGCC GSCs based on log2‐fold change.

#### 2.5.2 Protein‐mRNA correlation

Protein per‐gene expression was calculated as the average of the proteins' levels for those proteins matching to the same gene, whereas the mRNA per‐gene expression was calculated as the sum of TPMs per gene. Correlations between matching protein and mRNA expression levels per overlapping genes were tested with Spearman's correlation coefficient and two‐sided *t* test at *α* = 0.05 and corrected for multiple testing with the FDR. Protein‐mRNA correlation for the CPTAC data was performed using processed and normalized proteomic and transcriptomic data available from [6]. The selected gene sets for correlation analyses were extracted from the Molecular Signature Database (MSigDb) [35, 36], apart from the ‘Glioma‐elevated’ and ‘FDA drugs’ datasets, which were extracted from the Human Protein Atlas [37].

The Bland–Altman analysis on agreement in mRNA‐protein correlations between GSCs and GBM tissue was performed as previously described [38]. The genes outside the 95% CI (Confidence Intervals) of the Bland‐Alman plot were considered to have strong disagreement; we extracted the gene lists above and below the 95% CI and performed enrichment analysis with an overrepresentation test in g:Profiler.

#### 2.5.3 Features reduction, visual projection, and clustering

Principal component analysis (PCA), Uniform Manifold Approximation and Projection (UMAP), and hierarchical clustering of samples based on protein expression was performed on log2 relative protein expression values. We used the prcomp, umap, and Heatmap functions from the stats, umap, and ComplexHeatmap packages, respectively.

#### 2.5.4 ssGSEA, GSEA and MSigDB

Single‐sample gene set enrichment analysis (ssGSEA) was performed by ordering the protein rank according to their log2 relative protein expression values in a sample and performing a gene set enrichment analysis (GSEA) on gene sets of interest, adjusting for multiple comparisons at 5% FDR. For subtyping the GSCs, the Verhaak gene sets were downloaded from the MSigDB [35, 36] and we created a dataset with Entrez IDs for the Wang gene sets and the GSAPS protein sets. GSEA analyses were performed separately for published, hallmark (H), and GO (gene‐ontology) biological processes' gene sets by sub‐setting the MSigDB to the C2‐CGP (chemical and genetic perturbations), C2‐REACTOME, H, and C5 GO biological processes categories. The ranking in the comparisons GPC‐like vs. GM‐like GSCs and recurrent vs. primary GBM tissue was based on the difference in log2 average expression in the first group and the log2 average expression in the second group. In instances where several proteins matched to the same gene name, we excluded those proteins from the ranked GSEA list, to reduce uncertainty in the ranking. For all the GSE analyses we used the GSEA function from the clusterprofiler package.

#### 2.5.5 Protein–protein interactions

The known and predicted protein–protein interactions of the proteins included in the GSAPS were derived from string, v.11.5 [39]. The network was based on confidence values above 0.4 of the entire string network, including all data sources. The proteins were then further clustered with *k*‐means, assigning 10 clusters.

#### 2.5.6 GBM anatomical localization

Glioblastoma differentially expressed gene sets per anatomic region were downloaded from the Ivy League GBM Atlas [40], including gene sets of leading edge (*n* = 1998), cellular tumor (*n* = 114), palisades around necrosis (*n* = 389), and microvascular proliferation (*n* = 1126). The gene sets per regions consisted of genes two‐folds (log2‐FC > 1) differentially expressed in that region as compared to the remaining regions, at 1% FDR, based on an edgeR analysis. For the overlapping genes identified in our proteomic experiment and included in the Ivy GBM Atlas gene sets, we calculated the mean protein log2‐FC between GPC‐like and GM‐like HGCC GSCs as a difference between mean log2 protein values and categorized them as up in GPC‐like (if log2‐FC > 0) and up in GM‐like (if log2‐FC < 0). We then made contingency tables and tested if the proteins were overrepresented in the anatomical regions' gene sets with a two‐sided Fisher's exact test, at *α* < 0.05.

#### 2.5.7 CPTAC proteomics dataset

We downloaded a processed, mass‐spectrometry, global‐proteomics, log2‐normalized protein expression matrix of GBM tissue samples (*n* = 99) and normal brain tissue samples (*n* = 10), along with clinical, subtype, molecular, and survival data from the CPTAC cohort multiomic datasets [6]. Based on the expression of proteins included in the GSAPS, the samples were clustered with PCA and hierarchical clustering (method: Euclidean distance). We then performed ssGSEA for the refined GPC‐like and GM‐like protein set, by ranking the proteins within a sample based on their log2 relative expression values and considered the protein set enriched and upregulated in the sample if the enrichment score was > 0, at *P* < 0.05 and 5% FDR.

The OS was calculated as the time from date of initial pathological diagnosis to date of death or date of loss to follow‐up. The OS could not be calculated for two patients due to missing data. GPC‐like and GM‐like sum scores were calculated by summing up the relative protein expression values of the proteins included in the refined GPC‐like and GM‐like protein set, respectively, and log2‐normalizing them. We then performed a survival analysis with Cox proportional hazards models and a likelihood ratio test at *α* = 0.05, adjusting the scores for age and sex. We did not adjust for MGMT promotor methylation status because of the large proportion of missing values for this variable in the CPTAC cohort (*n* = 62, 63.918%). To confirm the association between the refined GPC‐like and GM‐like sum scores and OS, we categorized the scores based on quartile expression values to high/medium (> first quartile) and low score (< first quartile) and performed KM survival analysis with a logrank test, at *α* = 0.05. Finally, we calculated a log2 ratio of the refined GPC‐like to the GM‐like sum score and performed survival analysis with Cox proportional hazards models and likelihood ratio test, adjusting for age and sex. We then categorized the GPC‐like/GM‐like ratio to high (> third quartile), medium (> first and ≤ third quartile), and low (≤ first quartile) and performed KM survival analysis with a log‐rank test, at *α* = 0.05.

#### 2.5.8 Protein structure models

Experimental models for the protein structure of HNRNPA2B1 were downloaded from the PDB database: 1X4B (nuclear magnetic resonance, residues 1–103) and 5WWG (X‐ray crystallography, residues 12–195). Because a full‐length protein structure is not available for the canonical isoform of HNRNPA2B1, we used the AlphaFold2‐predicted structure model of isoform B1 (AF‐P22626‐F1) for comparison to the non‐canonical protein structure. The protein structure of the canonical A2 isoform and the non‐canonical protein structure of the new isoforms of HNRNPA2B1 (310‐ and 390‐residues long) were predicted with AlphaFold2 [41], using a Python Jupiter Notebook within ColabFold [42].

#### 2.5.9 Software

All analyses were performed in r v.4.0.3, unless stated otherwise. Visualization of protein structures and sequence matching was performed in UCSF Chimera, v.1.16 (University of California, San Franscisco, CA, USA). The figure panels were created in Adobe Illustrator 2020. The graphical abstract was created in Biorender (Toronto, Ontario, Canada).

## 3 Results

### 3.1 Protein identification and GBM proteome subtyping

To extract a protein signature that recapitulates GSC‐specific features, we analyzed six primary GSCs (hereafter referred to as BT GSCs) with RNAseq and in‐depth proteomic profiling (Fig. 1A). Three GSCs were previously classified as expressing the classical subtype and three expressing the proneural subtype (Wang 2017 mRNA classification, Table S4) [20, 21, 43]. All samples were run in biological triplicates. To extract proteins that are consistently deregulated in GSCs but not in other brain‐derived cells, we included primary human healthy astrocytes with three technical replicates representing normal brain cells, and three biological replicates of the T98G human glioblastoma cell line, representing a non‐stem glioblastoma cell line (hereafter defined as non‐GSC brain cell lines). Across all samples, we identified 11 140 proteins, of which 9161 proteins (82.24%) had no missing values and were included in the analyses. This is, so far, the most in‐depth proteomic characterization of GSCs.

Based on total proteome expression, the GSCs clearly clustered away from non‐GSC brain cell lines (Fig. 1B; Fig. S1A, Table S5). Performing ssGSEA to define the Verhaak GBM subtype at protein level showed that three cell lines overexpressed a different subtype at protein level compared to their initial mRNA subtype classification; some GSCs initially classified as proneural had enrichment for the classical subtype and vice versa. In addition, the proteins included in the proneural gene set projected closer to the classical gene set, suggesting that they were coexpressed in the GSCs (Fig. 1C,D). This also implied that classical GSCs are more closely related to the proneural GSCs in human samples, as suggested in a mouse cell‐of‐origin gene signature in mouse GSCs [23]. As expected, based on their original classification, the GSCs showed higher expression of protein products of genes included in both the proneural and classical subtype as compared to the non‐GSC lines, but had a consistently lower expression of proteins deriving from mesenchymal gene sets (Fig. S1B). Based on ssGSEA, we did not detect an activation of the refined Wang proneural and classical gene sets [5] at protein level, possibly because these gene sets are smaller than the Verhaak GBM gene sets [4]. However, all GSCs had a suppression for the Wang mesenchymal subtype, in agreement with the Verhaak gene sets (Fig. S2A,B). Furthermore, the MET gene had consistent downregulation in BT GSCs, and all GSCs had higher EGFR to MET ratio compared to non‐GSC cell lines (Fig. S2A,C), suggesting that higher EGFR‐to‐MET ratio and a downregulated MET could be biomarkers of the non‐mesenchymal subtypes. The downregulation of MET in classical GSCs agrees with previous findings [20], however, we found that MET is downregulated in proneural GSCs at protein level opposing the findings of De Bacco et al. [20].

This shows that whereas the cell lines have an interchangeable enrichment for the proneural and classical subtypes, they had a consistent suppression of the mesenchymal subtype.

### 3.2 Protein‐mRNA correlations in GSCs

**Fig. 2:**
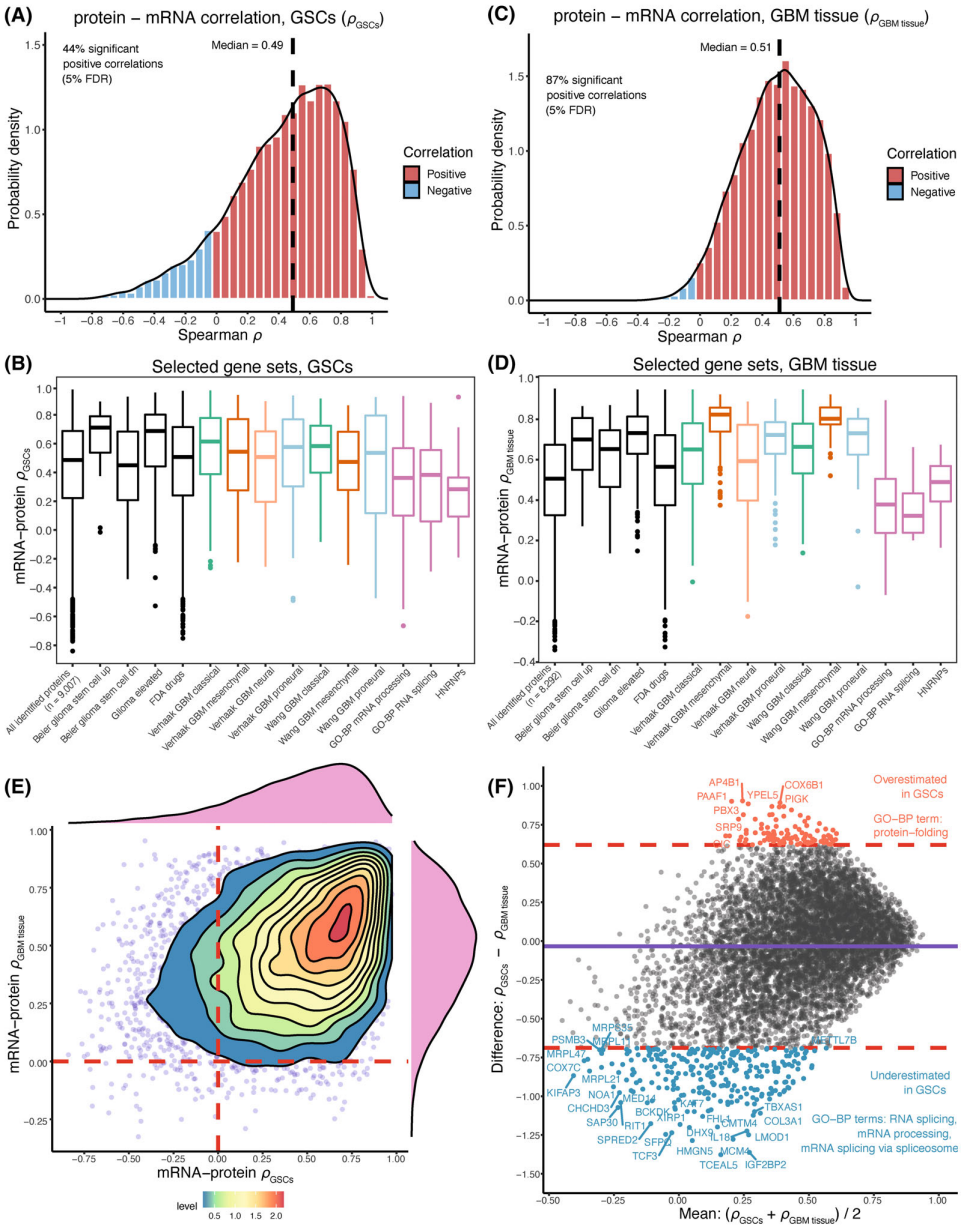
mRNA‐protein correlations in BT GSCs and in CPTAC GBM tissue. (A) mRNA‐protein Spearman correlation coefficients (*ρ*_*GSCs*_) of genes identified in BT GSCs (*n*_cell_ = 18) with both RNA‐seq and HiRIEF LC–MS/MS; (B) *ρ* *_GSCs_* of genes included in selected gene sets of interest; (C) mRNA‐protein correlation of genes (*ρ*_*GBM tissue*_) identified in GBM tissue, CPTAC cohort (*n*_tissue_ = 99); (D) *ρ* *_GBM tissue_* of genes included in selected gene sets of interest; (E) Density plot comparing mRNA‐protein correlation coefficients in GSCs and GBM tissue. Most of the genes had a positive correlation (*ρ* > 0) in both GSCs and GBM tissue; (F) Bland–Altman plot comparing the agreement between *ρ*_*GSCs*_ and *ρ*_*GBM tissue*_. The mean of the coefficients is plotted on the *x* axis and the difference between the coefficients is plotted on the *y* axis. The dashed lines show the 95% confidence intervals for the differences in correlation coefficients. Outside of the dashed lines are the genes with the largest disagreement in mRNA‐protein correlations in GBM tissue and GSCs. The proteins below the lower dashed line had significantly lower mRNA‐protein correlation in GSCs and proteins above the upper dashed line had significantly higher mRNA protein‐correlation in GSCs, as compared to GBM tissue. These genes' lists were enriched for the annotated gene ontology (GO) terms; the full enrichment terms are given in Fig. S3.

Due to the relatively low agreement between protein and mRNA expression levels in the gene sets used to classify GBM subtypes in the BT GSCs, we analyzed the overall agreement between mRNA and protein per‐gene products in GSCs. Per‐gene correlation analysis of mRNA and protein matching to 9007 genes showed an overall moderate agreement between mRNA and protein levels (median Spearman's *ρ* = 0.49, *P* < 0.05, 5% FDR, Fig. 2A; Table S6). Analyzing several established GBM and splicing gene sets of interest [4, 5, 37, 44] showed similar mRNA‐protein correlations as observed in the entire proteome identified in the GSCs (Fig. 2B). Genes upregulated in CD133+ glioma stem cells compared to CD133− glioma stem cells [44] and glioma‐elevated genes (obtained from the Human Protein Atlas – HPA [37]) had a higher than overall mRNA‐protein correlation, whereas genes involved in splicing and heterogeneous ribonucleoproteins (HNRNPs) had lower than overall correlation in GSCs.

To verify whether the overall moderate mRNA‐protein correlations are observable at GBM tissue level as well, we downloaded proteomic and transcriptomic data from the recently published CPTAC GBM cohort, which includes multiomic profiling of 99 treatment‐naïve GBM cancer tissues [6]. Based on an analysis of mRNA and protein products deriving from 8292 genes, GBM tissue also had a moderate overall mRNA‐protein correlation (median Spearman's *ρ* = 0.51, *P* < 0.05, 5% FDR, Fig. 2C; Table S7). GBM tissue had more statistically significant correlations and less skewed distribution of mRNA‐protein correlations compared to the GSCs, which is most likely due to the larger sample size of the GBM cohort that provided better estimates. The selected gene sets of interest showed mRNA‐protein correlation patterns in tissue like those in GSCs (Fig. 2D,E). Comparing the agreement between correlations' estimates by a Bland–Altman plot analysis showed that proteins involved in RNA splicing/processing and protein‐folding had lower and higher mRNA‐protein correlation in the GSC lines compared to GBM tissue, respectively (Fig. 2F; Fig. S3), suggesting that GSCs might have an impaired regulation of RNA metabolism but are less likely to accumulate unfolded proteins than GBM tissue due to better translation of proteins that regulate protein folding.

The mesenchymal gene sets had the highest concordance between mRNA and protein level in GBM tissue, with median correlation of *ρ* = 0.823 and *ρ* = 0.803 for the Verhaak and Wang mesenchymal gene sets, respectively (Fig. 2D). This was much higher compared to the median mRNA‐protein correlation in GSCs of *ρ* = 0.544 and *ρ* = 0.474 for the Verhaak and Wang mesenchymal gene sets, respectively (Fig. 2B). The classical and proneural gene sets also had higher mRNA‐protein correlation in GBM tissue, compared to GSCs, confirming that these gene sets might perform better at subtyping GBM tumors than subtyping GSCs. However, one limitation in our study is that the discovery panel did not include mesenchymal GSCs, which has limited the variance in protein levels for the subtypes' gene sets, albeit a minor effect (Fig. S4). It is also possible that non‐cancerous cells, such as stromal and immune cells, could have contributed to a larger variance in protein expression of genes included in the GBM subtypes, suggesting that the GBM subtypes expression patterns might not be fully reflected at GSC level. The higher correlations in tissue for the mesenchymal gene sets are expected, because it has been recently shown that this subtype has a larger infiltration of immune cells [6]. Still, other factors, such as gene sets' size, mRNA decay, protein degradation, study sample size, protein identification and technical measurement errors could have all contributed to the disagreement in estimating mRNA and protein correlations in both GSCs and GBM tissue.

Overall, our findings demonstrate that the regulation of mRNA translation to protein follows similar patterns in GSCs as in GBM tissue, and that GSCs can be a representative cell model for protein expression in GBM to some degree. However, there was a notable disagreement between mRNA and protein levels, which warrants investigating the GSCs at the phenotypic level by analyzing the proteome.

### 3.3 GSC‐associated protein signature reflects the proneural‐mesenchymal axis

**Fig. 3:**
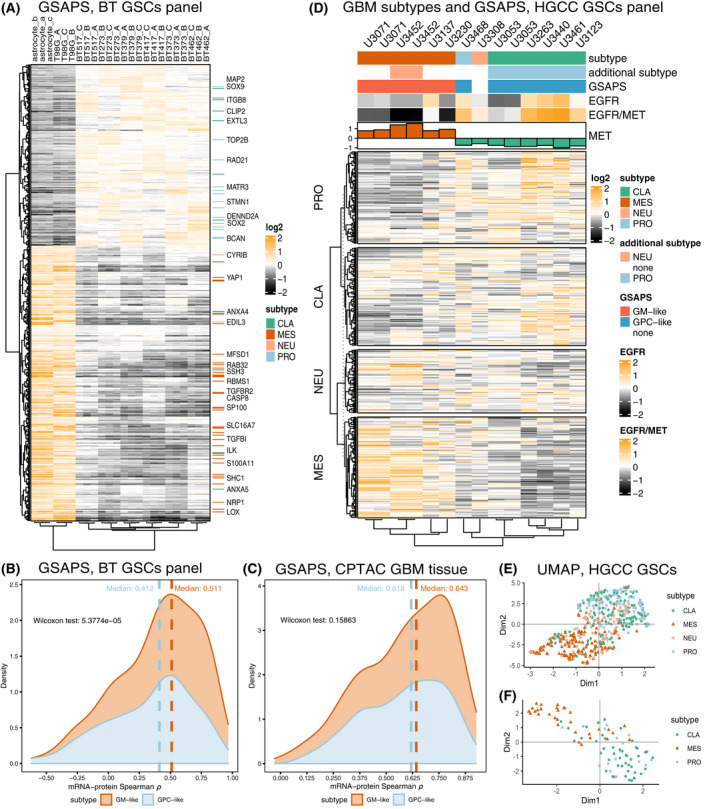
GSAPS expression in the HGCC panel of GSCs. (A) Hierarchical clustering of the BT GSC panel (*n* = 18) and non‐GSC cell lines (*n* = 4) with proteins included in GSAPS (distance: 1 − Spearman's *ρ*); (B) Correlation between protein and mRNA levels of GSAPS protein sets in GSCs; (C) Correlation between protein and mRNA levels of GSAPS protein sets in GBM tissue; (D) Hierarchical clustering based on the protein expression levels of genes included in the Verhaak gene sets (distance: 1 − Spearman's *ρ*), and EGFR and MET protein expression in GSCs of different subtypes; (E, F) UMAP dimensional reduction of the genes included in the Verhaak GBM subtypes' gene sets (E) and the revised Wang GBM subtypes' gene sets (F).

To select a set of proteins that describe GSC phenotypes, we performed a differential expression analysis, to define a GSC‐associated protein signature (GSAPS). We compared each GSC triplicate to each non‐GSC triplicate (astrocyte or T98G), to encompass the defining stem expression signature of each cell line and selected the overlapping proteins that were consistently differentially altered in the same direction in all BT GSCs and in all comparisons (Fig. S5). This led to a core set of 524 proteins that we define as GSAPS (Fig. 3A; Table S8). STRING analysis of the GSAPS proteins showed that most of them interact between each other (Fig. S6, Table S9A,B).

As expected, GSEA of GSAPS showed upregulation of the Verhaak proneural subtype gene set and downregulation of the mesenchymal subtype and the epithelial‐to‐mesenchymal transition (EMT) gene sets (Fig. S7, Tables S10–S12). Gliomas are not tumors derived from the epithelium, therefore the EMT is not directly applicable to them. However, a similar process, proneural‐to‐mesenchymal transition (PMT), has been described in GBM and is associated with worse prognosis and therapy resistance [5, 45, 46, 47, 48, 49]. A predominant part of GSAPS consisted of upregulated proneural and downregulated mesenchymal markers, suggesting that the GSAPS shows inverse association with PMT. The GSAPS also had the *hallmark* hypoxia gene set downregulated, along with several other hypoxia gene sets (Fig. S7B, Table S11). The hypoxic metabolism has been associated with the mesenchymal subtype [49, 50], suggesting that GSAPS can reflect the subtype‐driven cellular metabolic condition.

Based on the differences in gene sets enriched among the upregulated and downregulated GSAPS proteins, we split it into two protein sets. The first consisted of the upregulated GSAPS proteins associated with the proneural signature and proliferation, henceforth referred to as GSAPS Proneural and Classical‐like protein set (GPC‐like), and the other consisted of the downregulated GSAPS proteins, associated with the mesenchymal signature, hypoxia, and EMT, henceforth referred to as GSAPS Mesenchymal‐like protein set (GM‐like). We hypothesized that these two protein sets define two different GSC conditions, which are mutually exclusive and would better define the specific GSC phenotypes than the previously established Verhaak and Wang gene signatures established for GBM tissue. Worth noticing is that 107 of the GSAPS proteins are targetable by FDA‐approved drugs (31 in the GPC‐like and 76 in GM‐like set, Table S13), with some drugs targeting more than one protein in the signature and 33 drugs ongoing clinical trials in GBM (Table S14).

The overall protein‐mRNA correlation of genes encoding for the GSAPS proteins was moderately positive (Spearman's median *ρ* = 0.459), indicating that some features should be detectable at mRNA level but a considerable proportion of the GSC phenotype variance will be observable only at protein level. We detected a higher mRNA‐protein agreement for genes included in the GM‐like set in GSCs (Fig. 3B), but this was not observed in GBM tissue (Fig. 3C), which had higher mRNA‐protein agreement for the GSAPS sets than GSCs.

### 3.4 GSAPS defines two phenotypic conditions

To address the lack of mesenchymal GSCs in the first GSC panel, and to confirm the GSAPS ability to define GSC conditions along the PMT axis, we performed proteomic expression profiling on another GSC panel consisting of GSCs of all GBM subtypes. The extended cohort included 11 patient‐derived GSC lines from the HGCC cohort, identifying 10 169 proteins across the cell lines, including cell lines classified as mesenchymal based on mRNA expression [9]. Subtyping the cell lines with ssGSEA at protein level showed that all GSCs that expressed the classical subtype also expressed the proneural subtype and had a suppression for the mesenchymal subtype (Fig. 3D), in line with previous observations on BT GSCs. Clustering the proteins corresponding to the subtype‐specific genes included in Verhaak [4] and Wang [5] GBM gene sets showed again that the proteins included in the classical gene set projected closer to the proteins included in the proneural gene set and apart from the mesenchymal proteins (Fig. 3E,F).

**Fig. 4:**
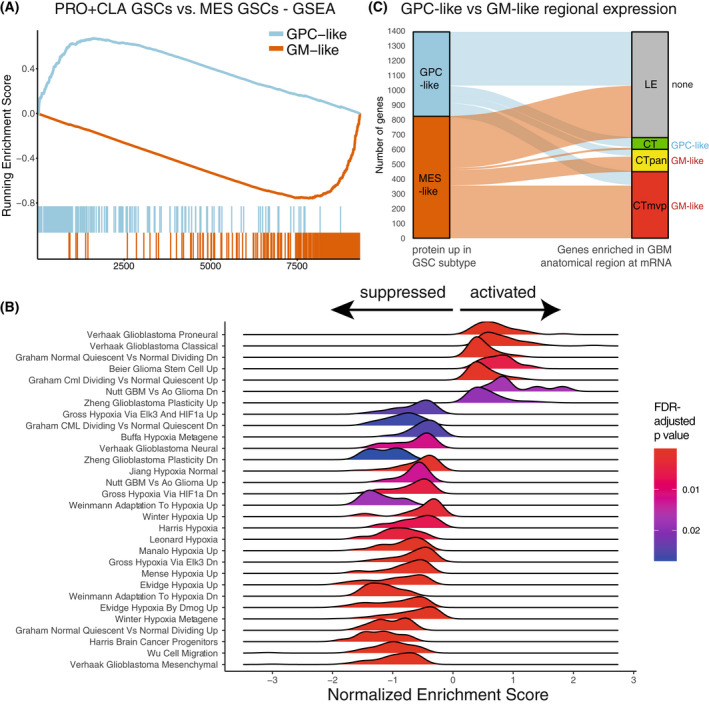
Proneural‐mesenchymal axis and GSAPS association with different gene sets and pathways. (A) GSEA of the GSAPS protein sets GPC‐like and GM‐like in proneural and classical GSCs as compared to mesenchymal GSCs. The GPC‐like and GM‐like protein sets were enriched in the proneural and classical GSCs and mesenchymal GSCs, respectively (NES > 3, *P* < 0.001, 1% FDR); (B) Selected MSigDb C2 gene sets enriched in the GPC‐like GSCs as compared to GM‐like GSCs at *P* < 0.05 (two‐sided permutation test) and 5% FDR, GSEA, *x* axis = normalized enrichment score; (C) Sankey diagram depicting the proportion of genes upregulated in the GPC‐like or GM‐like GSCs at protein level, based on log2‐FC only, which is enriched in different anatomical regions of GBM: leading edge (LE), cellular tumor (CT), cellular tumor palisading around necrosis (CTpan), and cellular tumor's microvascular proliferation (CTmvp). On the right side of the diagram, the enriched protein set is annotated per region (two‐sided Fisher's exact test, *P* < 0.001, 1% FDR).

Applying GSAPS to the HGCC panel clustered the mesenchymal GSCs separately from proneural‐classical GSCs (Fig. S8). To further validate whether the GSAPS is reflective of PMT, we performed GSEA on the two GSAPS protein sets comparing the proneural‐classical HGCC GSC lines to the mesenchymal GSC lines. As hypothesized, we detected a strong enrichment of both GSAPS protein sets (NES > 3, *P* < 0.001), with the GPC‐like set upregulated in the proneural‐classical GSCs and the GM‐like upregulated in the mesenchymal GSCs (Fig. 4A,B). ssGSEA analyses showed that GSCs expressing the GPC‐like phenotype had suppression of the GM‐like phenotype, and vice versa, confirming the hypothesis that these conditions are mutually exclusive. GSEA comparing the protein expression of GPC‐like GSCs to GM‐like GSCs on *hallmark* gene sets showed that GM‐like GSCs were enriched for the hypoxia gene set (Fig. S9).

Considering that mesenchymal gene expression has been consistently associated with hypoxia, we hypothesized that the GM‐like GSCs could be enriched in hypoxic regions of GBM tumor tissue, in proximity to necrosis, such as regions of tumor cells palisading around necrosis (CTpan) and tumor cells involved in microvascular proliferation (CTmvp). We then performed enrichment analysis comparing protein expression between GPC‐like and GM‐like GSCs to genes enriched in different GBM anatomical regions at mRNA level, based on the Ivy GBM Atlas [40], which consisted of genes enriched at mRNA level in different GBM regions. Neither GSAPS set had enrichment in the leading edge (LE) region of GBM. However, genes with higher protein levels in the GM‐like GSCs were enriched in regions of CTmvp and CTpan, whereas genes with higher protein levels in GPC‐like GSCs were enriched in regions of cellular tumor – CT (Fig. 4C). The findings suggest that GSCs adapt their phenotypic expression and thereby their subtype to local conditions, driving different elements of tumorigenesis. This is in line with previous observations within the Ivy GBM Atlas [40]. Still, the gene sets of the Ivy Atlas are derived by transcriptomic methods, leaving a gap to explore the regional protein expression in GBM for future endeavors.

To address the limitations in the first GSC panel, which lacked GSCs of the mesenchymal subtype and included a comparison to non‐GSC cell lines that were grown in serum‐containing media that could have potentiated the “mesenchymalness” of cells *in vitro* [51], we further refined the GSAPS in the HGCC GSCs. For this purpose, we filtered the GPC‐like protein sets to include only proteins that were upregulated in the proneural‐classical HGCC GSCs (*n* = 157, 75.845%) and the GM‐like protein set to include only proteins that were upregulated in the mesenchymal HGCC GSCs (*n* = 256, 81.270%) based on log2‐fold change (Table S15). This refined GSAPS was used for all subsequent analyses in GBM tissue.

In summary, these findings confirm that GSAPS is associated with PMT and that cultured GSCs exist in two mutually exclusive phenotypic conditions, one characterized by the GPC‐like protein set and another characterized by the GM‐like protein set. These two types of GSCs have an inverse association with hypoxia, with GM‐like GSCs having a higher activation of hypoxia‐induced gene sets compared to GPC‐like GSCs.

### 3.5 GSAPS is enriched in recurrent GBM tissue

**Fig. 5:**
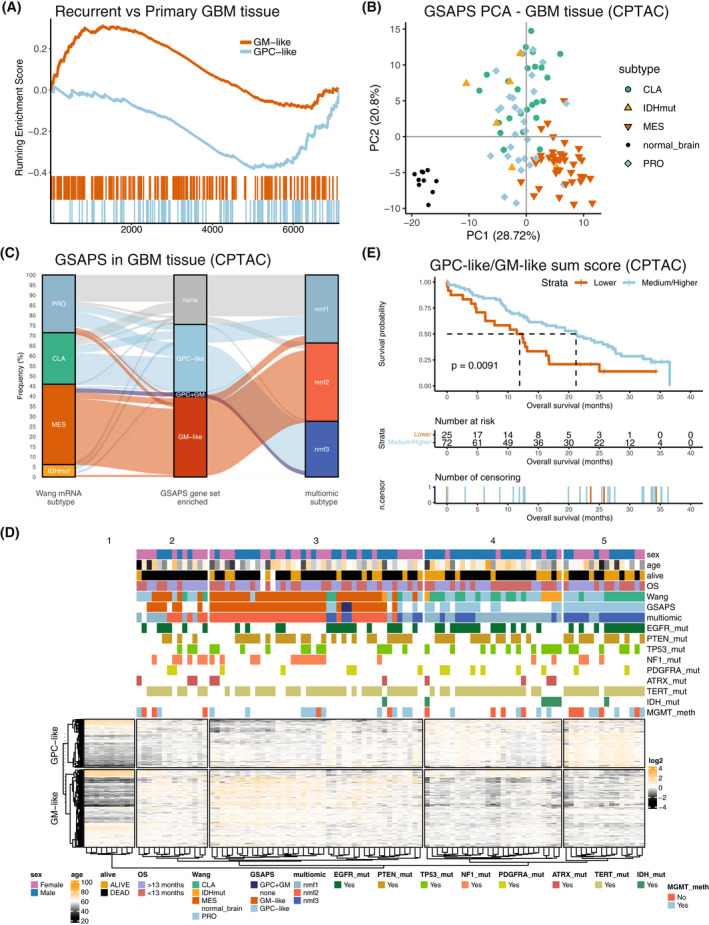
Refined GSAPS expression in GBM tissue. (A) GSEA on the GSAPS protein sets GPC‐like and GM‐like comparing recurrent to primary GBM tissue tumors (*P* < 0.001, 1% FDR); (B) PCA clustering, based on log2 expression levels of proteins included in the GSAPS, of GBM tumors and normal brain tissue samples. GBM subtypes (mRNA, based on the Wang 2017 GBM classification [5]): CLA, classical; PRO, proneural; IDHmut, IDH‐mutant tumor; MES, mesenchymal; (C) Sankey diagram showing the proportion of GBM tumors of different transcriptomic subtypes (Wang 2017, GBM classification) that are enriched for the GSAPS protein sets GPC‐like or GM‐like or both, as compared to the CPTAC's multiomic GBM subtypes recently described by Wang et al. [6]; (D) Hierarchical clustering of GBM tumors and normal brain samples based on the GSAPS (distance: 1 − Spearman's *ρ*). The different subtypes are shown in the annotation bars, as well as mutation status of common genomic markers in GBM; (E) KM curves showing survival differences in patients categorized based on log2 GPC‐like to GM‐like protein sum score ratio to groups of low (≤ first quartile) and medium/high (> first quartile) score ratios. The *P*‐values are based on logrank tests; the dashed lines present the median overall survival in the corresponding groups.

Recurrent GBM tumors tend to have worse outcome and faster progression. Several studies have linked this to PMT, suggesting that proneural and classical GSCs are more sensitive to chemotherapy and radiotherapy, which eventually leads to selection and enrichment of the mesenchymal subtype within recurrent tumors [5, 49]. To test whether GM‐like GSCs are enriched in recurrent GBM tumors, we analyzed seven primary and three recurrent GBM tissue samples at proteomic level with HiRIEF LC–MS/MS, identifying 7810 proteins, with 7378 proteins quantified in all samples. GSEA between non‐paired recurrent and primary tumors showed activation of the mesenchymal GBM gene set and suppression of pathways associated with GPC‐like GSCs in recurrent tumors (Fig. S10). As hypothesized, GSEA on the GSAPS gene sets, comparing recurrent to primary GBM, showed a suppressed GPC‐like and activated GM‐like protein set in recurrent GBM tumors (Fig. 5A). The GM‐like protein set was also associated with necrosis (Fig. S11).

### 3.6 GSAPS protein signatures are associated with OS in GBM tissue

A GSAPS‐expression PCA analysis of 99 GBM tumors and 10 normal brain samples from the CPTAC cohort [6] separated GBM from normal brain tissue and separated mesenchymal from non‐mesenchymal cancer tissue (Fig. 5B–D). Considering that the GM‐like signature was associated with hypoxia, necrosis, and recurrence in GBM tissue, we hypothesized that it might be associated with worse OS in GBM. To prove the hypothesis, we calculated GPC‐like and GM‐like protein sum scores by summarizing relative expression of the proteins included in the corresponding refined GSAPS protein sets and performed survival analyses. Adjusting for sex and age, which was associated with worse OS in this GBM cohort, higher GPC‐like and GM‐like protein sum scores on their own had a statistically non‐significant association with longer and shorter OS, respectively (Table S16, Fig. S12A,B). To incorporate both protein sets, we then calculated a log2 ratio of the GPC‐like to GM‐like protein sum score (log2 GPC‐like/GM‐like), which showed that higher GPC‐like/GM‐like ratios were associated with better OS (HR = 0.463, 95% CI: 0.224–0.957, LRT, *P* = 0.005), adjusted for age and sex in Cox models. This association remained consistent by categorizing log2 GPC‐like/GM‐like ratio to quartile expression in KM curves (Fig. 5E, log‐rank test, *P* = 0.0091). The association was inversed when calculating a log2 GM‐like/GPC‐like ratio (Fig. S12C,D).

Overall, these results show that GSAPS describes a GSC cellular signal that can categorize tumors across the PMT axis, and that lower protein expression of the GPC‐like signature combined with a higher protein expression of the GM‐like signature was associated with worse OS in GBM.

### 3.7 New protein‐coding targets in GSCs

Stem cells often utilize parts of the genome that mature cells do not, such as early developmental genes, to obtain pluripotency. To explore if GSCs express non‐canonical proteins, i.e., proteins deriving from genome regions considered as non‐protein‐coding, we employed a previously established proteogenomics pipeline [32, 34], to search for non‐canonical protein sequences in BT GSCs. For this aim, we created an RNAseq‐based database of predicted protein sequences, by translating the detected transcript sequences obtained from RNAseq to protein sequences, further predicting corresponding tryptic peptides by *in silico* tryptic cleavage. We then appended the non‐canonical database to a canonical database of protein sequences and searched for non‐canonical peptides among the identified PSMs. This approach allowed us to discover novel non‐canonical peptides matching to protein sequences corresponding to genome regions predicted to be non‐protein‐coding, such as pseudogenes and lncRNAs, as well as non‐canonical peptides matching to protein‐coding genes that have not been previously described, such as novel start sites, splice variants, gene extensions, etc.

**Fig. 6:**
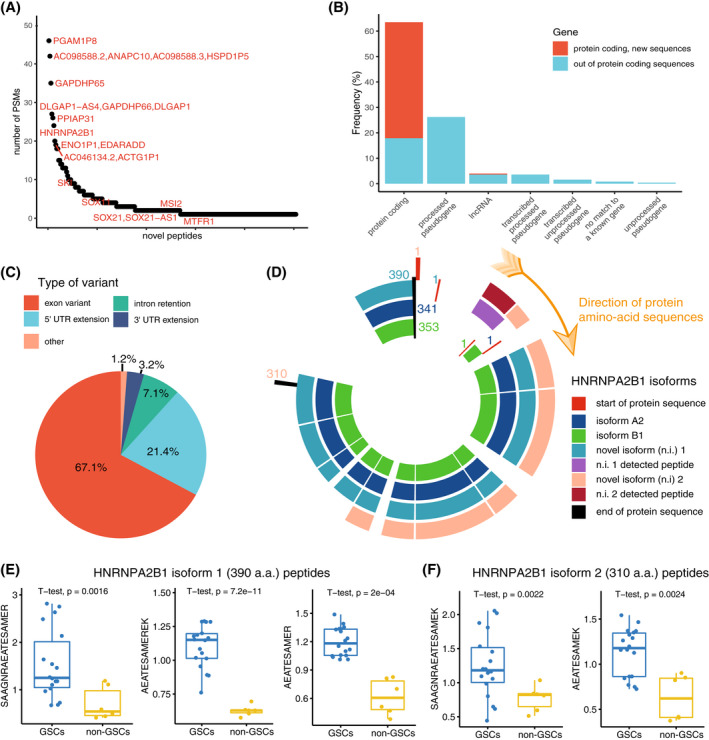
Non‐canonical peptides identified in the discovery panel of cell lines (*n* = 22). (A) Number of PSMs per non‐canonical peptide; (B) Proportion of non‐canonical and novel peptides classified according to matching gene type; (C) Proportion of novel peptide classified according to the matching gene region; (D) Canonical (A2 and B1) and non‐canonical protein isoforms (here referred to as n.i. 1 and n.i. 2, both include 5′ extensions) of the HNRNPA2B1 gene. The plot shows the detected peptides positioned to the matching sequences of the canonical and novel isoforms of the HNRNPA2B1 gene. The numbers refer to the positions of the first and last amino acid of the corresponding isoform; (E) Boxplots comparing levels of novel peptides matching to the novel protein isoform 1 of HNRNPA2B1 (390 amino acids long), two‐sided *t* test. The whiskers' limits are the minimum and maximum values of the data after removing outliers; (F) Boxplots of novel peptides matching to the novel protein isoform 2 of HNRNPA2B1 (310 amino acids long), two‐sided *t* test.

We detected 252 non‐canonical peptides in BT GSCs, half of them with two or more PSMs (*n* = 118, 53.17%, Fig. 6A; Table S17). More than half (53.97%) were novel peptides, whereas the remaining peptides matched to non‐canonical sequences of protein‐coding genes (Fig. 6B,C). One tenth of the non‐canonical peptides (*n* = 23) matched to protein‐coding genes included in the GSAPS, as expected – mostly the GPC‐like protein set (*n* = 19), including exon variants of HNRNPA2B1, QKI, CUX1, EPHB3 and GAB1, and 5′‐UTR extensions of SOX2, TRIM24, QKI, and MSI2.

A recent screen of non‐canonical open‐reading frames characterized hundreds of new proteins in human induced pluripotent stem cells and human foreskin fibroblasts [52]. To validate the novel peptides discovered in our study, we downloaded the novel amino‐acid sequences reported by Chen et al., and found that 40 of the non‐canonical peptides discovered in our study overlapped with the non‐canonical protein sequences reported by the authors, providing independent support (Table S17). Most of these non‐canonical peptides were extensions of protein‐coding genes (*n* = 33, 82.5%).

### 3.8 Heterogeneous nuclear ribonucleoproteins' variants contain 5′‐UTR peptide sequences

Sixteen non‐canonical peptides found in GSCs matched to a family of the ubiquitously expressed HNRNPs, which are involved in mRNA splicing, processing, and metabolism [53]. Half of these peptides matched to processed pseudogenes (HNRNPA1‐P8, ‐P12, ‐P14, ‐P16, and ‐P59), and the remaining half to variants of the isoforms A2 and B1. Among the non‐canonical peptides matching to protein‐coding genes, several matched to two novel protein‐coding isoforms of HNRNPA2B1 reported by Chen et al. [52], which have upstream extensions of the canonical protein isoforms' sequences (Fig. 6D). The canonical HNRNPA2B1 protein had a higher expression in BT GSCs compared to non‐GSC lines and was part of the GPC‐like protein set, along with other HNRNPs (HNRNP‐U, ‐D, ‐DL, and ‐LL). Interestingly, the non‐canonical peptides matching to the HNRNPA2B1 gene also had higher levels in BT GSCs compared to non‐GSC lines (Fig. 6E; Table S18, *P* < 0.05, 5% FDR), suggesting a role in GSC biology. Still, it remains to be elucidated if the non‐canonical protein sequences detected in GSCs in this study, such as those of HNRNPA2B1, exist only in GSCs or provide improved gene models overall.

Finally, we used AlphaFold2 [41] to explore whether protein expression of the 5′‐UTR region in the novel isoforms of HNRNPA2B1 could alter protein structure. Although with low confidence, the AlphaFold2 structure prediction of the 390‐residues‐long novel isoform of HNRNPA2B1 suggests that expression of a 5′‐UTR sequence might form alpha helices that are not predicted from the canonical sequence of isoforms A2 and B1 or observed in experimental models (Fig. S13). We speculate that such an alteration in protein structure can affect the protein function and lead to the formation of new epitopes, potentially recognizable by the immune system.

Overall, our findings show that some gene variants previously considered as non‐coding are expressed and translated in GSCs and GBM at protein level and that a subset of these is related to proteins included in the GSAPS.

## 4 Discussion

Glioblastoma is a highly malignant cancer, driven by GSCs and their ability to adapt in response to treatment and the tumor microenvironment. To improve treatment options for GBM patients, it is essential to understand the underlying mechanisms driving GSCs and how mRNA is translated to protein level, allowing the tumor to progress, adapt, and resist therapeutic interventions.

In this study, we have performed the most in‐depth proteogenomic analysis of GSCs to date, providing an extensive, new data resource for GSC proteome expression. This data resource provides a new layer of information on GSC biology, which we believe will be valuable for future studies on gene expression and translation to proteins in GSCs. Derived from a comparison of primary proneural and classical GSCs to non‐GSC brain cell lines, we present a new GSC‐associated protein signature. Some of the GSAPS protein products derive from genes included in the gene sets that define the GBM subtypes. Thus, we named the GSAPS protein sets GPC‐like and GM‐like according to the overlap with and enrichment of the corresponding GBM subtypes' gene sets. However, worth pointing out is that GSAPS is not synonymous with the GBM subtypes gene sets, but instead the GPC‐like and GM‐like protein sets describe two phenotypic GSC conditions that are mutually exclusive, reflecting the proneural‐to‐mesenchymal axis, and having an inverse association with necrosis, recurrence, and OS in GBM. Although mesenchymal GSCs were not included in the derivation of GSAPS, this did not curb the identification of the GM‐like protein set, which was strongly suppressed in GSCs enriched for proneural and classical markers. We further showed that this exclusive expression of the GSAPS protein sets is strongly preserved in GSCs by analyzing another panel of GSCs from the HGCC cohort [9], where mesenchymal GSCs had a strong activation of the GM‐like and suppression of the GPC‐like protein set. Furthermore, we showed that at protein level GBM proneural and classical gene sets are both enriched in non‐mesenchymal GSCs, which had a strong activation of the GPC‐like and suppression of the GM‐like protein set. We further used the HGCC GSC panel to refine the signature, to filter out protein signals that could have been driven by the protein expression in non‐GSC brain cells, which served as comparison. We used the T98G cell line as a glioblastoma cell line that is a tumor non‐stem cell line, and the astrocyte line as a normal brain cell line, to represent non‐GSC brain cells, because the cellular proteome of these cells is more comparable than brain tissue due to the presence of extracellular matrix and connective fibers, which would affect the protein levels. The choice of two non‐stem cell lines for comparison might have limited the analysis but using several lines for comparison might lead to a trade‐off of a highly specific GSC signature that would maybe lack in sensitivity. However, our analysis of normal brain tissue samples used in the CPTAC cohort showed that neither of the GSAPS protein sets was enriched in normal brain tissue, and the majority of the GSAPS proteins had lower expression in normal brain tissue as compared to GBM tissue. Still, it is not unexpected that normal brain cells would share some protein expression with brain tumor cells, as observed in single‐cell RNA‐sequencing studies, which, for example, identified GBM tumor cells with astrocyte‐like and oligodendrocyte precursor cell‐like transcriptome signatures [19].

By analyzing protein expression in GBM cancer tissue, we show that the refined GM‐like protein set was associated with recurrent GBM tissue, necrosis, and mesenchymal GBM tumors. Previous observations at tissue proteome level from the CPTAC cohort [6] have shown that mesenchymal GBM tumors have higher MET levels and are enriched for EMT, hypoxia, glycolysis, angiogenesis, and inflammatory pathways. We demonstrated that all these observations at tissue level are driven by expression patterns at GBM cellular level, delineating expression patterns deriving from the cancer cells in GBM tissue. Although the GSCs were not cultured in hypoxic conditions, the GM‐like GSCs had higher levels of proteins involved in regulating hypoxia compared to GPC‐like GSCs. This does not imply that either of the GSC types were hypoxic, but that they have a different expression of genes involved in regulating hypoxia. From a clinical perspective, the GSAPS encompasses over 100 protein drug targets, out of which 33 are currently undergoing clinical trials for GBM. It is tempting to speculate that the signature might serve a purpose in drug development guidance, where a combination treatment targeting proteins in both the GPC‐like and the GM‐like protein set might be more effective. Furthermore, the GSAPS demonstrated prognostic value, where lower GPC‐like over GM‐like ratios were associated with worse OS in GBM. Considering that existing transcriptomic signatures were not predictive of OS in GBM [4, 5], this discovery of a GSC‐associated protein signature that can predict OS is worth of further exploration in larger, independent cohorts.

Proteogenomics allows for the discovery of non‐canonical protein sequences that have not been observed before, matching to new protein variants, or pseudogenes and long‐non‐coding RNAs not expected to be protein coding. Through a proteogenomic approach, we discovered protein sequences matching to genes previously established as non‐protein coding, where some of these have a higher expression in GSCs. Our unique approach provides a more confident discovery of non‐canonical protein sequences because it involves verification of the matching sequences at both mRNA and protein level, detected by RNAseq and HiRIEF LC–MS/MS, respectively. The identification of non‐canonical proteins in GSCs questions established gene models and indicates potentially new proteins, which may have implications in GBM and warrant further investigation. AlphaFold2 predictions of the protein structure of an HNRNPA2B1 isoform that expresses protein sequences from the 5′‐UTR region suggest that non‐canonical proteins might have an altered protein structure, which could hypothetically create new epitopes recognizable as non‐self by the immune system. Such protein variants and novel peptides open a door for immunotherapy development for GBM, serving as starting point for development of immunotherapies with cancer vaccines and CAR T‐cells.

## 5 Conclusions

In summary, we present an in‐depth proteogenomic characterization of GSCs and report a new GSC‐associated protein signature that differentiates two phenotypic conditions of GSCs along the proneural‐to‐mesenchymal axis. This signature was associated with cancer aggressiveness in patients with GBM, including OS. Furthermore, in a unique approach, we discover novel protein‐coding gene regions in GSCs, which may have implications in GBM biology and potential treatment development. Our findings allow studying GBM at a GSC cellular proteomic level, improve our understanding of GSC biology, and identify new, protein‐ and pathway‐related, subtype‐specific therapeutic targets for GSCs.

## Conflict of interest

The authors declare no conflict of interest.

## Author contributions

HB, SG, SAC, JL, and MP conceptualized and designed the study. SG, DC, SP, AMi, and SAC prepared the discovery panel of GSCs. NM, MH, and LU provided the HGCC panel of GSCs. GT and AMa provided the GBM tissue samples. HB prepared the samples for proteomics LC–MS/MS analysis, analyzed the data, wrote the initial draft of the manuscript, and prepared the figures. HMU performed the proteogenomic identification. SG, SAC, JL, and MP provided funding, supervised the work, and were involved in writing the initial draft of the manuscript. All authors were involved in corrections of the initial draft and approved the manuscript draft.

## Supporting information


**Fig. S1.** Protein expression of GSC markers described in literature.
**Fig. S2.** Protein expression of genes included in the Wang GBM subtypes' gene sets.
**Fig. S3.** g:Profiler enrichment analysis of the genes that were outside of the 95% confidence intervals (CI) of the Bland–Altman plot comparing the agreement in mRNA‐protein correlation estimates in GBM tissue and GSCs.
**Fig. S4.** Relation between per‐gene mRNA‐protein correlations of GBM subtypes' gene sets in GSCs and GBM tissue, and variance of protein expression of the corresponding GBM gene sets.
**Fig. S5.** Differential expression algorithm for detecting GSAPS.
**Fig. S6.** STRING analysis of protein–protein interactions of proteins included in the GSAPS.
**Fig. S7.** Gene set enrichment analysis (GSEA) of the initial GSAPS, at 5% FDR.
**Fig. S8.** Hierarchical clustering of HGCC GSCs based on initial GSAPS protein expression.
**Fig. S9.** Gene set enrichment analysis (GSEA) of hallmark gene sets from the MSigDB, comparing protein expression of GPC‐like GSCs to protein expression of GM‐like GSCs, at 5% FDR.
**Fig. S10.** Pathways enriched in recurrent vs. primary GBM tumors.
**Fig. S11.** Single‐sample GSEA of the refined GSAPS gene sets in the necrotic sample (p < 0.001, 1% FDR).
**Fig. S12.** Overall survival in GBM patients based on expression of the refined GSAPS, Kaplan–Meier (KM) curves, CPTAC data.
**Fig. S13.** Prediction of protein structure of canonical and non‐canonical isoforms of HNRNPA2B1 with AlphaFold2.

**Table S1.** Labelling scheme for three TMT‐10 sets for experiments of proteomic profiling of BT GSCs, the T98G line, and the astrocyte line.
**Table S2.** Labelling scheme for one TMT‐16 set for experiments of proteomic profiling of HGCC GSCs.
**Table S3.** Labelling scheme for one TMT‐10 set for experiments of proteomic profiling of GBM tumor tissue.
**Table S4.** Clinical data of patients that originated the 6 BT GSC panel (all untreated primary glioblastomas, WHO grade IV gliomas), and GSC classification.
**Table S5.** Common GSC markers used for hierarchical clustering in Fig. S1A.
**Table S6.** Correlation between mRNA and protein expression of genes in GSCs detected both with RNA‐sequencing and HiRIEF LC–MS/MS.
**Table S7.** Correlation between mRNA and protein expression of genes in CPTAC GBM tissue detected both with RNA‐sequencing and LC–MS/MS.
**Table S8.** Proteins included in the GSAPS signature, i.e., consistently differentially expressed in all the GSCs as compared separately to the astrocyte and T98G line.
**Table S9A.** STRING analysis of protein–protein interactions of proteins included in GSAPS.
**Table S9B.** STRING cluster analysis of GSAPS proteins based on k‐means, assigning 10 clusters.
**Table S10.** Gene set enrichment analysis of the proteins included in the GSAPS signature, gene sets included in the MSigDb C2 databases – REACTOME and GCP.
**Table S11.** Gene set enrichment analysis of the proteins included in the GSAPS signature, showing enriched gene sets included in the MSigDb H database (hallmark processes).
**Table S12.** Gene set enrichment analysis of the proteins included in the GSAPS signature, gene sets included in the MSigDb C5, GO (Gene ontology) biological processes.
**Table S13.** Proteins in GSAPS targetable by drugs, according to the DrugBank.
**Table S14.** Proteins in GSAPS targetable by drugs that are ongoing clinical trials for GBM, according to the DrugBank.
**Table S15.** Proteins included in the refined GSAPS signature – filtering out proteins that had a different direction of deregulation in the HGCC GSCs based on log2‐fold change, compared to the log2‐fold change in the BT GSCs.
**Table S16.** CPTAC data used for survival analyses. Age, sex, BMI, tumor size, and Wang and multiomic subtypes (as classified by the CPTAC consortium) are included.
**Table S17.** Non‐canonical peptides discovered with a proteogenomic analysis of the BT GSCs, with annotation which match to non‐canonical proteins reported in Chen et al. (2020).
**Table S18.** Differentially altered novel peptides between the GSCs (n = 18) compared to controls – T98G and astrocyte line (n = 6).

## Data Availability

The mass spectrometry proteomics data are deposited to the ProteomeXchange Consortium via the PRIDE partner repository with the dataset identifiers: PXD027341, PXD027339, and PXD027335. RNAseq files are deposited to the SRA database, with the dataset identifier PRJNA886110. The code used for the analyses can be provided by the first or corresponding authors upon reasonable request.
