# Supplementary material for: Glioblastoma stem cells express non‐canonical proteins and exclusive mesenchymal‐like or non‐mesenchymal‐like protein signatures
Source: Mol Oncol. 2023 Jan 16;17(2):238–60. doi: 10.1002/1878-0261.13355 (PMC9892829; doi:10.1002/1878-0261.13355)
Supplement: Supplementary file 1 — Fig. S1. Protein expression of GSC markers described in literature. Fig. S2. Protein expression of genes included in the Wang GBM subtypes' gene sets. Fig. S3. g:Profiler enrichment analysis of the genes that were outside of the 95% confidence intervals (CI) of the Bland–Altman plot comparing the agreement in mRNA‐protein correlation estimates in GBM tissue and GSCs. Fig. S4. Relation between per‐gene mRNA‐protein correlations of GBM subtypes' gene sets in GSCs and GBM tissue, and variance of protein expression of the corresponding GBM gene sets. Fig. S5. Differential expression algorithm for detecting GSAPS. Fig. S6. STRING analysis of protein–protein interactions of proteins included in the GSAPS. Fig. S7. Gene set enrichment analysis (GSEA) of the initial GSAPS, at 5% FDR. Fig. S8. Hierarchical clustering of HGCC GSCs based on initial GSAPS protein expression. Fig. S9. Gene set enrichment analysis (GSEA) of hallmark gene sets from the MSigDB, comparing protein expression of GPC‐like GSCs to protein expression of GM‐like GSCs, at 5% FDR. Fig. S10. Pathways enriched in recurrent vs. primary GBM tumors. Fig. S11. Single‐sample GSEA of the refined GSAPS gene sets in the necrotic sample (p < 0.001, 1% FDR). Fig. S12. Overall survival in GBM patients based on expression of the refined GSAPS, Kaplan–Meier (KM) curves, CPTAC data. Fig. S13. Prediction of protein structure of canonical and non‐canonical isoforms of HNRNPA2B1 with AlphaFold2. [file MOL2-17-238-s001.pdf]

*Supplementary Figures to manuscript:*

Babačić H. *et al.* (2022). **Glioblastoma stem cells express non-canonical proteins and exclusive mesenchymal-like or non-mesenchymal-like protein signatures**

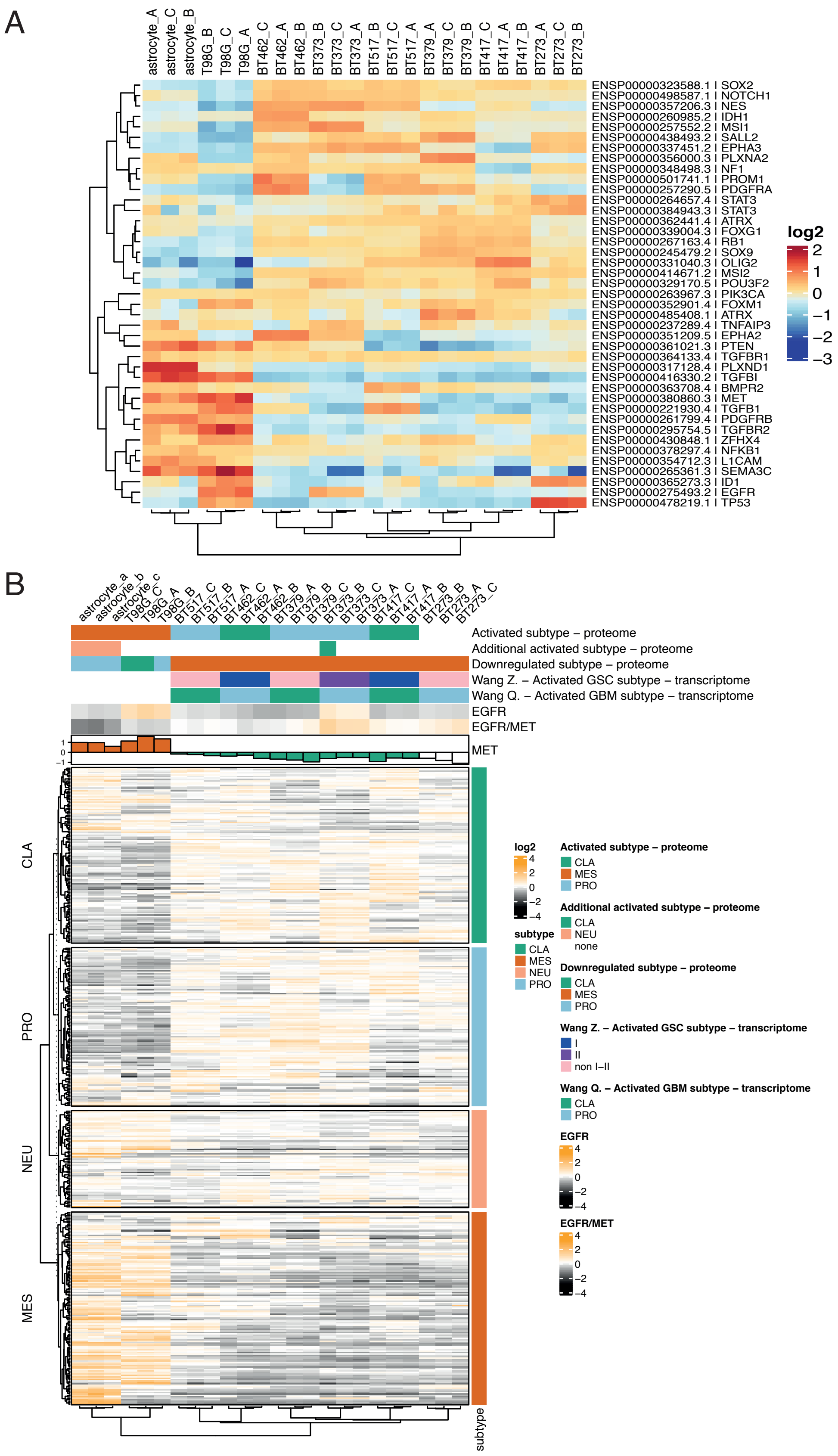


**Figure S1. Protein expression of GSC markers described in literature.**

**A.** Hierarchical clustering (distance: 1-Spearman's correlation coefficient) of GSCs and controls (astrocyte and T98G line) based on relative expression of known GSC

markers; **B.** Protein expression of genes included in the Verhaak (2010) GBM subtypes' gene sets identified in this study. Hierarchical clustering (distance: 1-Spearman's correlation coefficient) of GSCs based on protein expression of the gene sets. Annotation map – the Wang Q. refers to the GBM mRNA subtypes classification of the GSCs based on mRNA expression. Wang Z. classification refers to the recently proposed GSC classification to type I and type II (see DeBacco *et. al*, 2021).
Abbreviations: CLA = classical, PRO = proneural, MES = mesenchymal, NEU = neural.

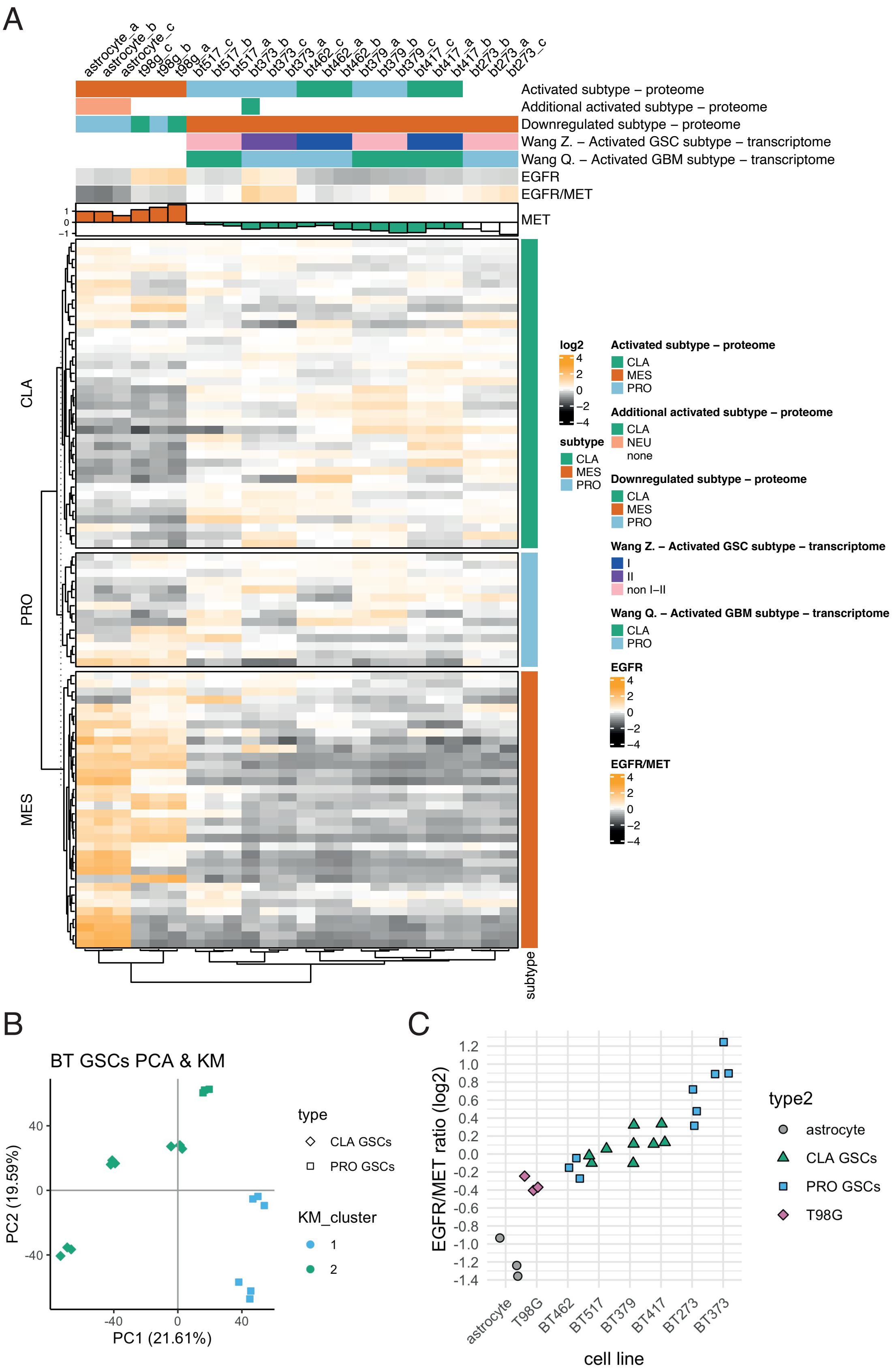


**Figure S2. Protein expression of genes included in the Wang GBM subtypes' gene sets. A.** Hierarchical clustering (distance: 1-Spearman's correlation coefficient)

of GSCs and controls (astrocyte and T98G line) based on relative expression of the Wang GBM subtypes' gene sets; **B.** For comparison - PCA and k-means (KM) clustering only of GSCs based on the expression of all the proteins without missing values. There was no clear separation between classical (CLA) and proneural (PRO) GSCs, as classified according to the Wang mRNA subtypes; **C.** EGFR/MET protein ratio on a log2 scale.

A

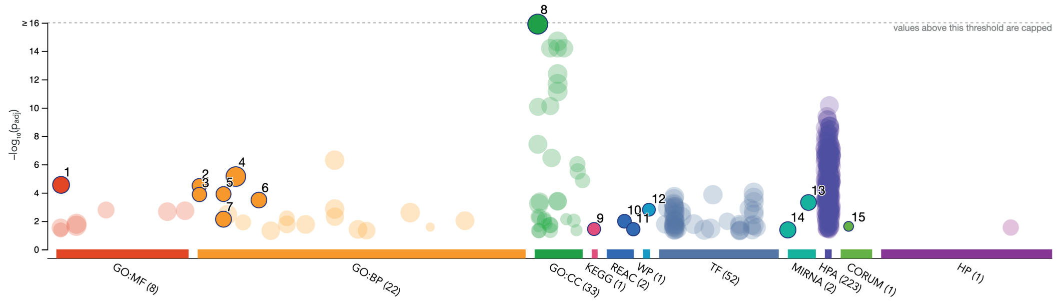


| ID | Source | Term ID | Term Name | $p_{adj}$ (query_1) |
|---|---|---|---|---|
| 1 | GO:MF | GO:0003723 | RNA binding | $2.799\times10^{-5}$ |
| 2 | GO:BP | GO:0000375 | RNA splicing, via transesterification reactions | $3.226\times10^{-5}$ |
| 3 | GO:BP | GO:0000398 | mRNA splicing, via spliceosome | $1.333\times10^{-4}$ |
| 4 | GO:BP | GO:0008152 | metabolic process | $7.242\times10^{-6}$ |
| 5 | GO:BP | GO:0006397 | mRNA processing | $1.248\times10^{-4}$ |
| 6 | GO:BP | GO:0016071 | mRNA metabolic process | $3.291\times10^{-4}$ |
| 7 | GO:BP | GO:0006396 | RNA processing | $7.423\times10^{-3}$ |
| 8 | GO:CC | GO:0005622 | intracellular anatomical structure | $1.253\times10^{-16}$ |
| 9 | KEGG | KEGG:03040 | Spliceosome | $3.654\times10^{-2}$ |
| 10 | REAC | REAC:R-HSA-7... | Processing of Capped Intron-Containing Pre-m... | $1.032\times10^{-2}$ |
| 11 | REAC | REAC:R-HSA-7... | mRNA Splicing - Major Pathway | $3.768\times10^{-2}$ |
| 12 | WP | WP:WP411 | mRNA Processing | $1.587\times10^{-3}$ |
| 13 | MIRNA | MIRNA:hsa-mi... | hsa-miR-615-3p | $4.912\times10^{-4}$ |
| 14 | MIRNA | MIRNA:hsa-let... | hsa-let-7b-5p | $4.196\times10^{-2}$ |
| 15 | CORUM | CORUM:1335 | SNW1 complex | $2.374\times10^{-2}$ |

**version** e104_eg51_p15_3922dba
**date** 16/12/2021, 18:16:39
**organism** hsapiens

g:Profiler

B

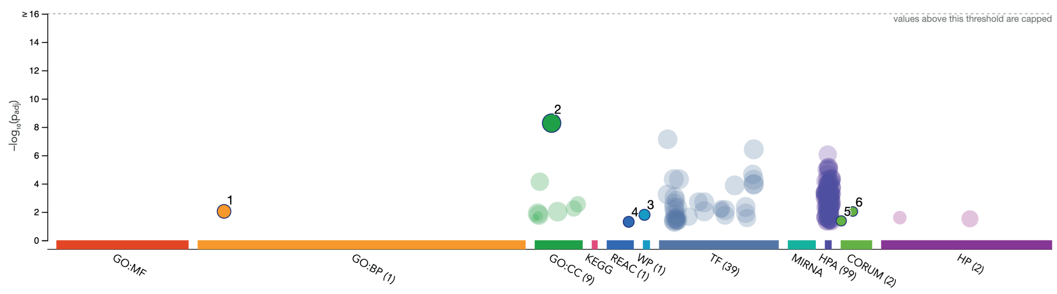


| ID | Source | Term ID | Term Name | $p_{adj}$ (query_1) |
|---|---|---|---|---|
| 1 | GO:BP | GO:0006457 | protein folding | $9.111\times10^{-3}$ |
| 2 | GO:CC | GO:0032991 | protein-containing complex | $5.324\times10^{-9}$ |
| 3 | WP | WP:WP405 | Eukaryotic Transcription Initiation | $1.596\times10^{-2}$ |
| 4 | REAC | REAC:R-HSA-5... | Signaling by FGFR2 in disease | $4.986\times10^{-2}$ |
| 5 | CORUM | CORUM:103 | RNA polymerase II holoenzyme complex | $4.264\times10^{-2}$ |
| 6 | CORUM | CORUM:2685 | RNA polymerase II (RNAPII) | $9.282\times10^{-3}$ |

**version** e104_eg51_p15_3922dba
**date** 16/12/2021, 18:15:09
**organism** hsapiens

g:Profiler

**Figure S3. g:Profiler enrichment analysis of the genes that were outside of the 95% confidence intervals (CI) of the Bland-Altman plot comparing the agreement in mRNA-protein correlation estimates in GBM tissue and GSCs. A.** Gene sets enriched in the list of proteins below the lower 95% CI, i.e., genes that had lower correlations in the GSCs compared to GBM tissue; **B.** Gene sets enriched in the list of proteins above the lower 95% CI, i.e., genes that had higher correlations in the GSCs compared to GBM tissue.

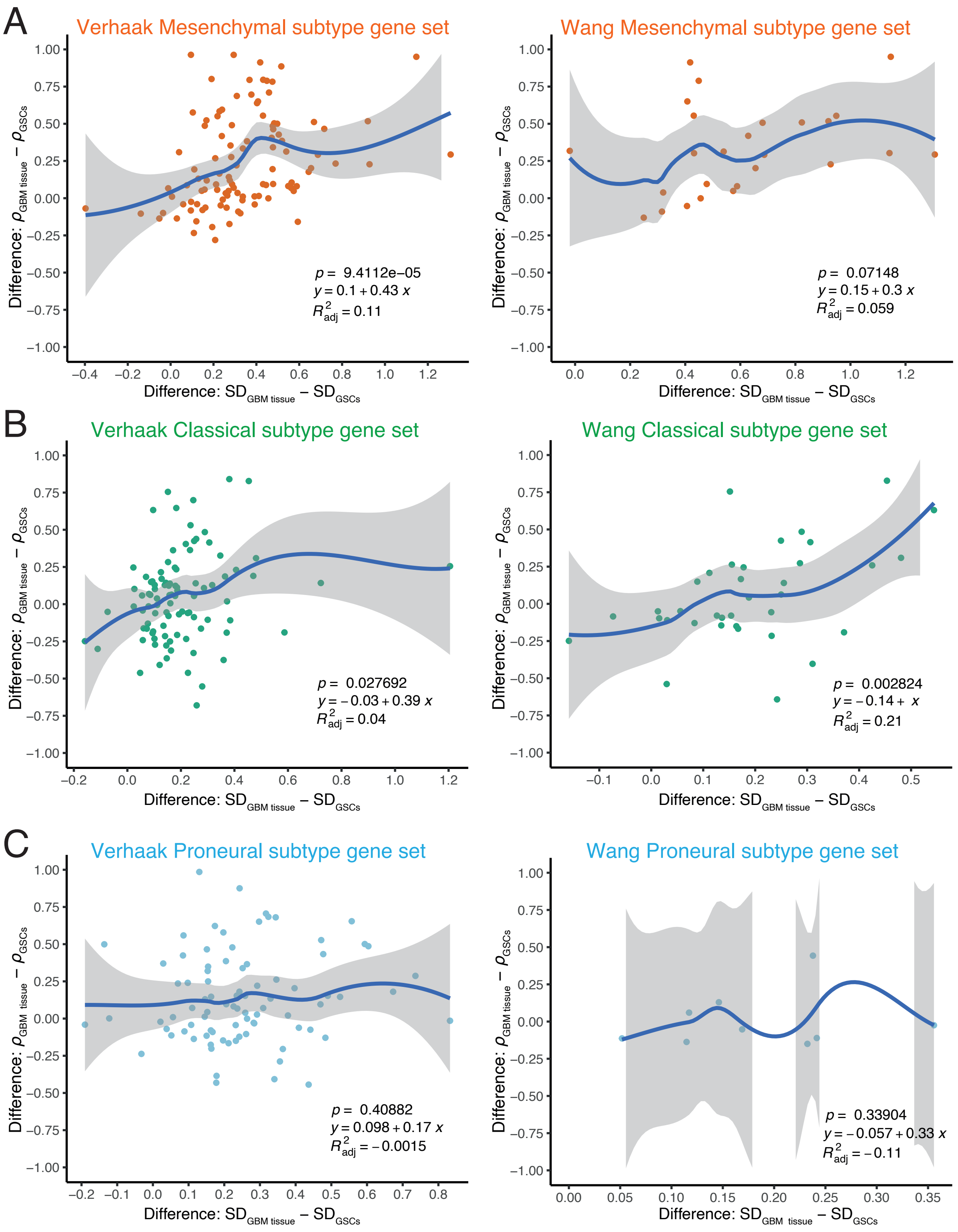


**Figure S4. Relation between per-gene mRNA-protein correlations of GBM subtypes' gene sets in GSCs and GBM tissue, and variance of protein expression of the corresponding GBM gene sets.** The difference between the standard deviations (SD) of protein expression in GBM tissue and GSCs (x axis) was associated with the difference in mRNA-protein correlations (Spearman correlation

coefficient - $\rho$) estimated in GBM tissue and in GSCs (y axis). Each dot represents a protein included in the respective gene set. Notice that the higher variance (SD) of protein expression in GBM tissue was associated with higher correlation coefficients in GBM tissue ($\rho_{GBM\ tissue}$) compared to GSCs ($\rho_{GSCs}$) for the Verhaak mesenchymal **(A)** and Verhaak and Wang classical **(B)** gene sets**,** but not for the proneural gene sets **(C)**. The equations refer to linear regression models explaining the difference in $\rho$ by the difference in SD. Although statistically significant, only a small proportion of the difference between $\rho_{GBM\ tissue}$ and $\rho_{GSCs}$ can be explained by the difference in the variance of protein expression between GBM ($SD_{GBM\ tissue}$) and GSCs ($SD_{GSCs}$).

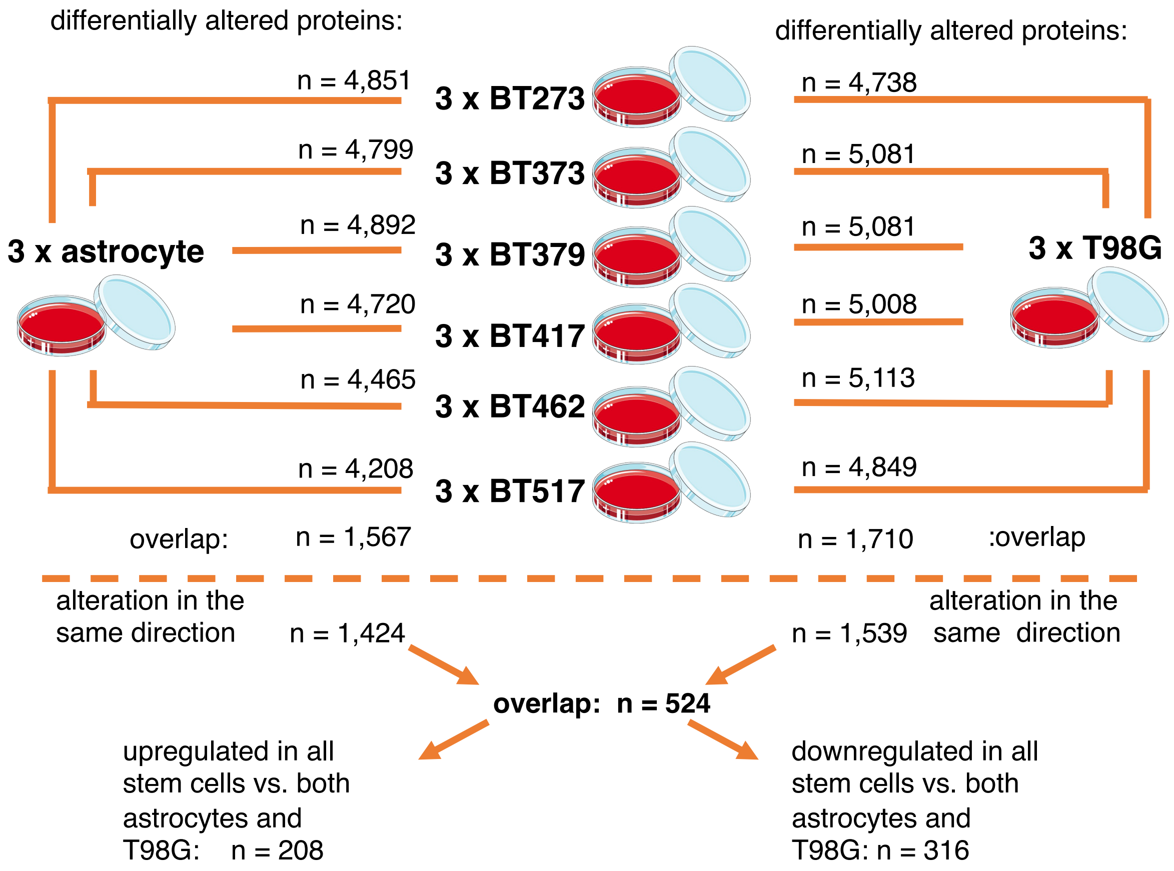


**Figure S5. Differential expression algorithm for detecting GSAPS.** Each GSC line was independently compared to the astrocyte and T98G line with a two-sided *t* test. Then we extracted the intersect of differentially expressed proteins in the same direction (over-/under-expressed in GSCs) in the comparison to the astrocyte and the T98G line, respectively. Finally, we took the intersect of consistently upregulated and downregulated proteins in GSCs that define GSAPS.

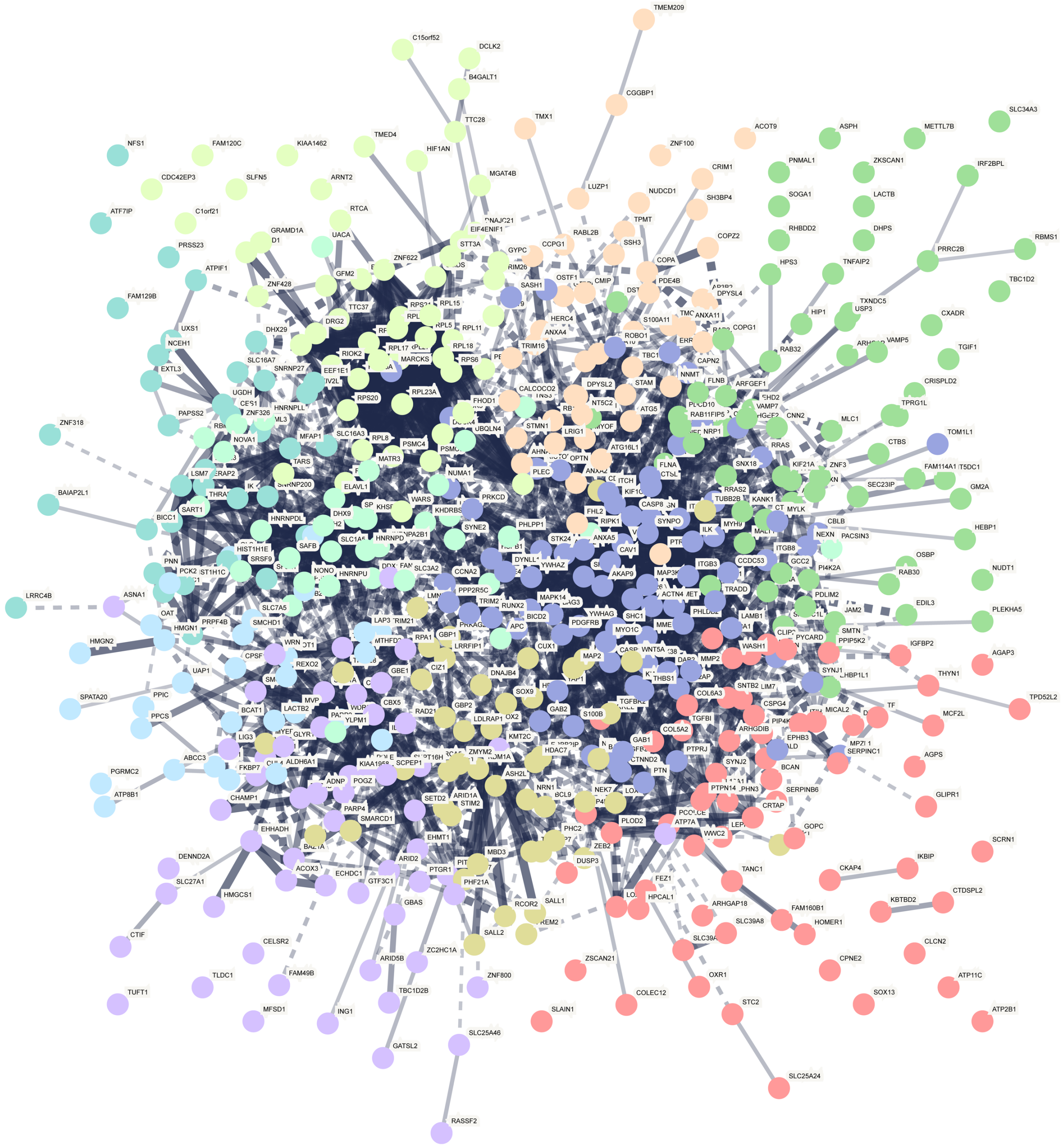


**Figure S6. STRING analysis of protein-protein interactions of proteins included in the GSAPS**. Up to 50 interactions are shown. Protein nodes are labelled according to their belonging to a protein cluster (k-means clustering, k = 10). See Supplementary Tables S9A, B for details.

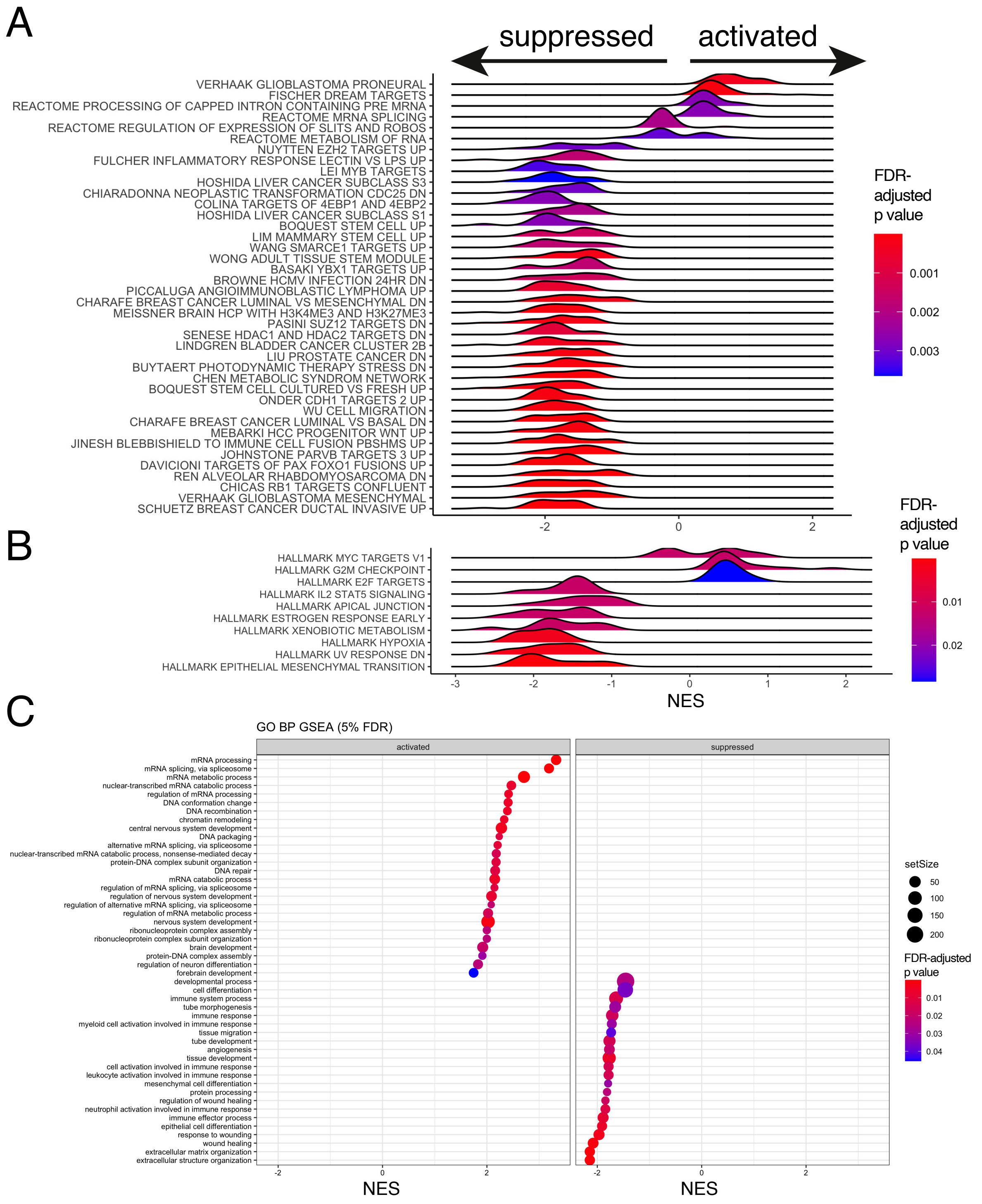


**Figure S7. Gene set enrichment analysis (GSEA) of the initial GSAPS, at 5% FDR. A.** GSEA of top enriched chemical and genetic perturbations (GCP) and REACTOME gene sets included in the C2 collection of gene sets in the Molecular Signatures Database (MSigDB); **B.** GSEA of hallmark gene sets included in the H collection of gene sets in the MSigDB; **C.** GSEA of selected GO biological processes (BP) terms. Abbreviations: NES = normalized enrichment score; FDR = false discovery rate.

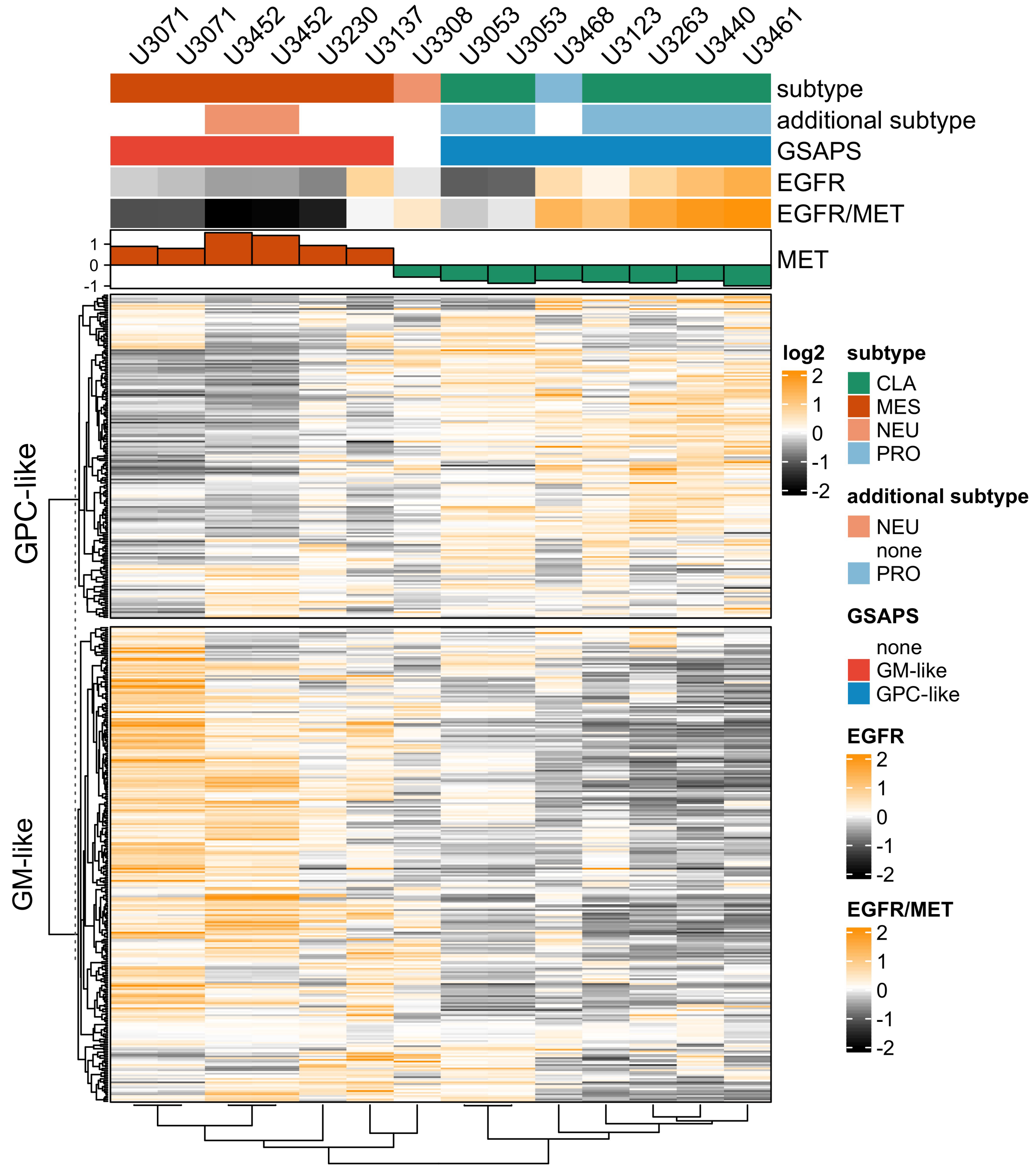


**Figure S8. Hierarchical clustering of HGCC GSCs based on initial GSAPS protein expression.** All classical GSCs had enrichment for the proneural subtype and the GPC-like GSAPS gene set, whereas the mesenchymal GSCs had enrichment for the GM-like gene set. Proneural (PRO) & classical (CLA) GSCs had a higher EGFR/MET ratio and lower MET levels, whereas the mesenchymal (MES) GSCs had a lower EGFR/MET ratio and higher MET levels. Abbreviations: NEU = neural.

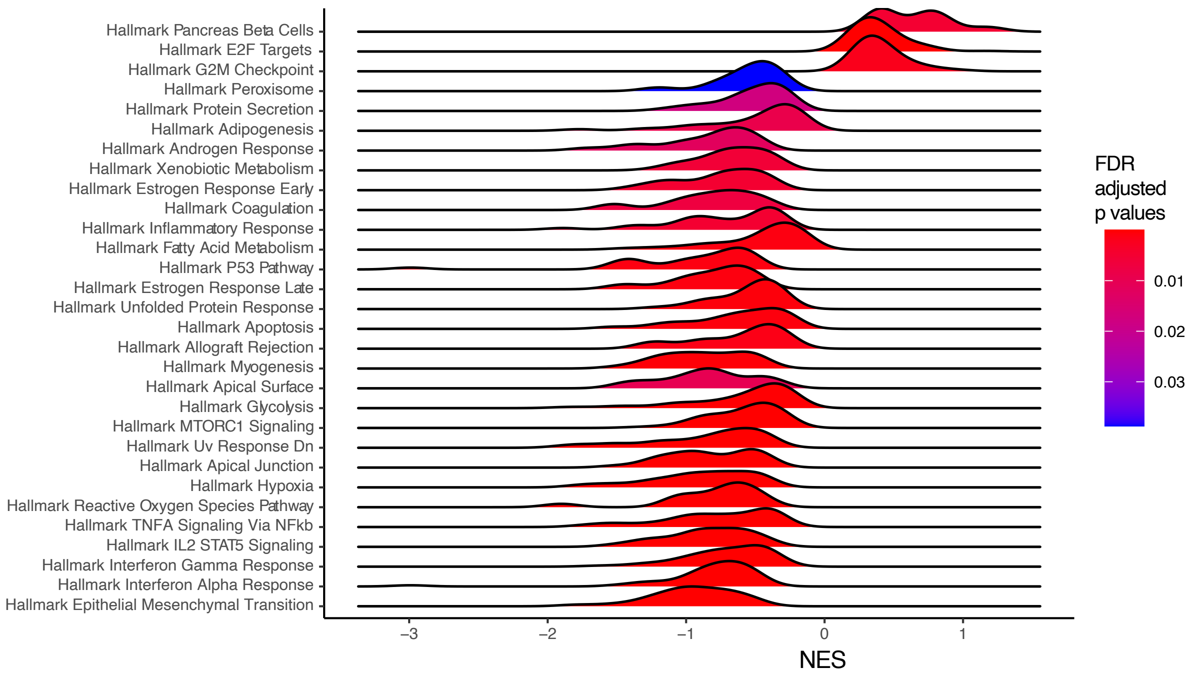


**Figure S9. Gene set enrichment analysis (GSEA) of *hallmark* gene sets from the MSigDB comparing protein expression of GPC-like GSCs to protein expression of GM-like GSCs, at 5% FDR.**

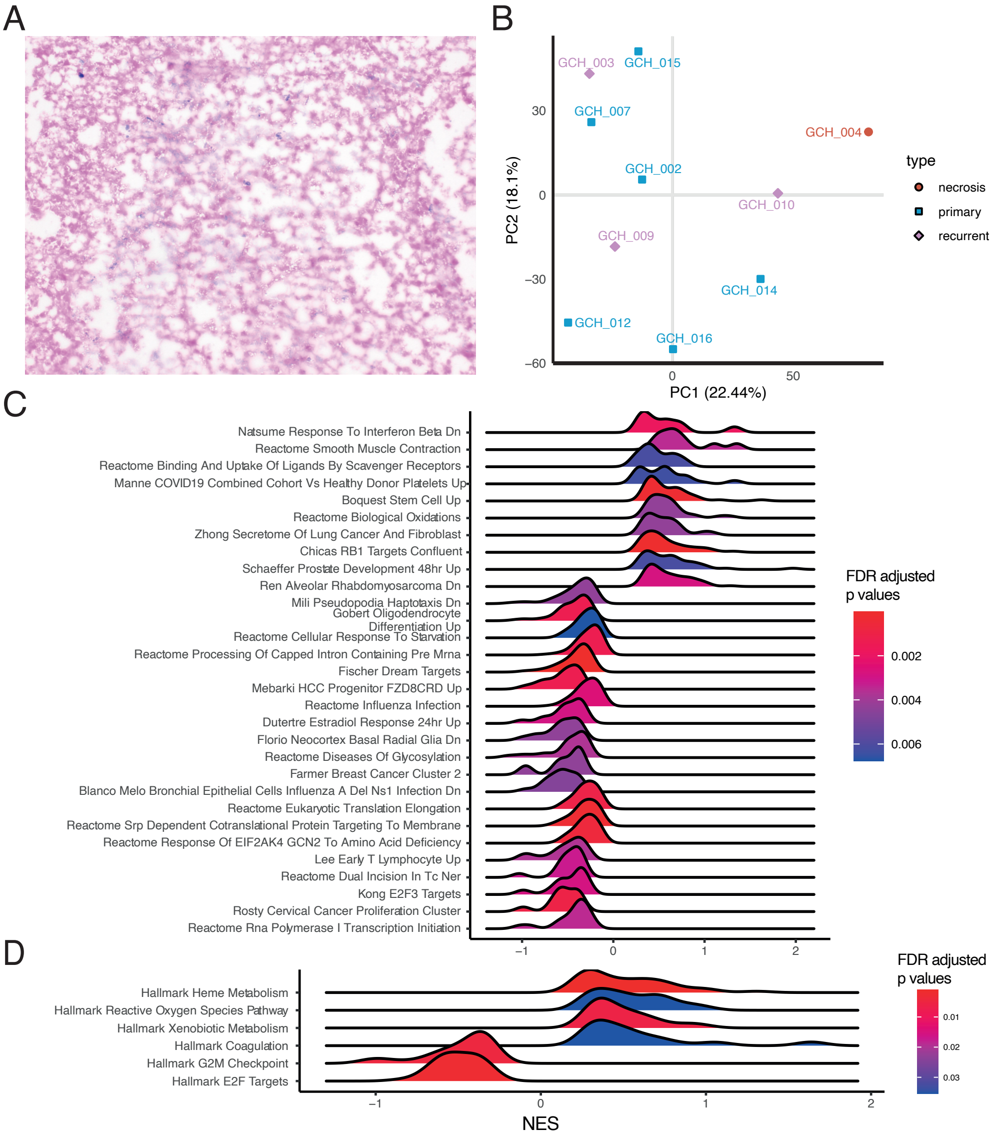


**Figure S10. Pathways enriched in recurrent vs. primary GBM tumors. A.** Hematoxylin and eosin staining of sample GCH004 showed extensive necrosis. The staining was repeated on another section and confirmed that the sample was necrosis. This sample was excluded from subsequent analyses; B. PCA clustering of GBM tissue samples based on bulk proteome expression; C. GSEA of top enriched C2 (subcategory: GCP and REACTOME) gene sets of the MSigDb, comparing recurrent to primary GBM tumors. The proteins were ranked based on a mean log2-FC comparing recurrent (n = 3) to primary (n = 6) GBM samples; D. GSEA of enriched hallmark (H) gene sets of the MSigDb, comparing recurrent to primary GBM tumors.

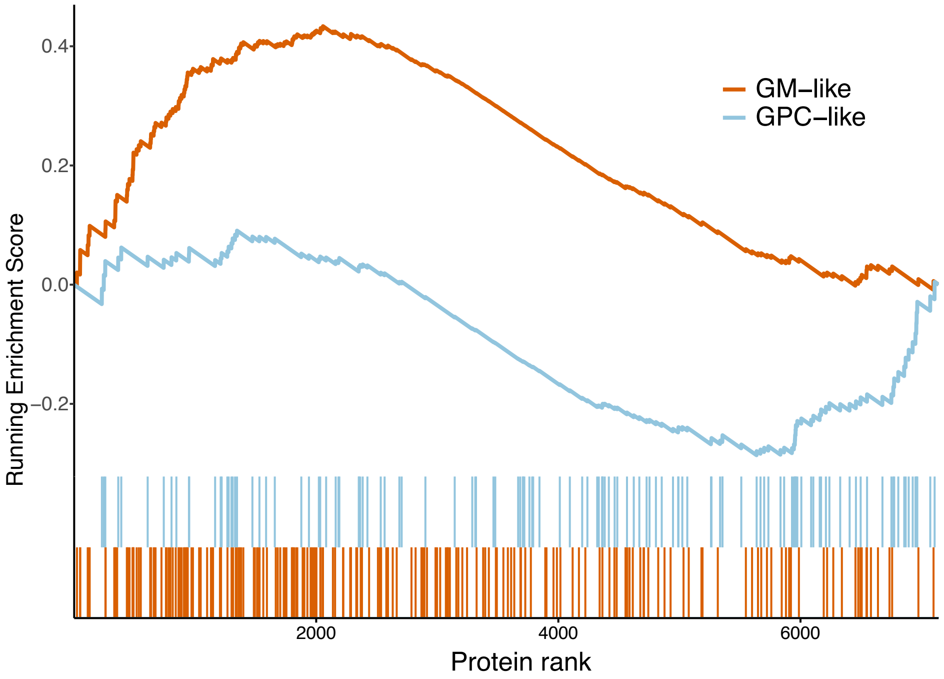


**Figure S11. Single-sample GSEA of the refined GSAPS gene sets in the necrotic sample ($p < 0.001$, 1% FDR).** Although the GM-like gene set was upregulated in the necrotic GBM tumor and the GPC-like was suppressed, the signals were not that consistent.

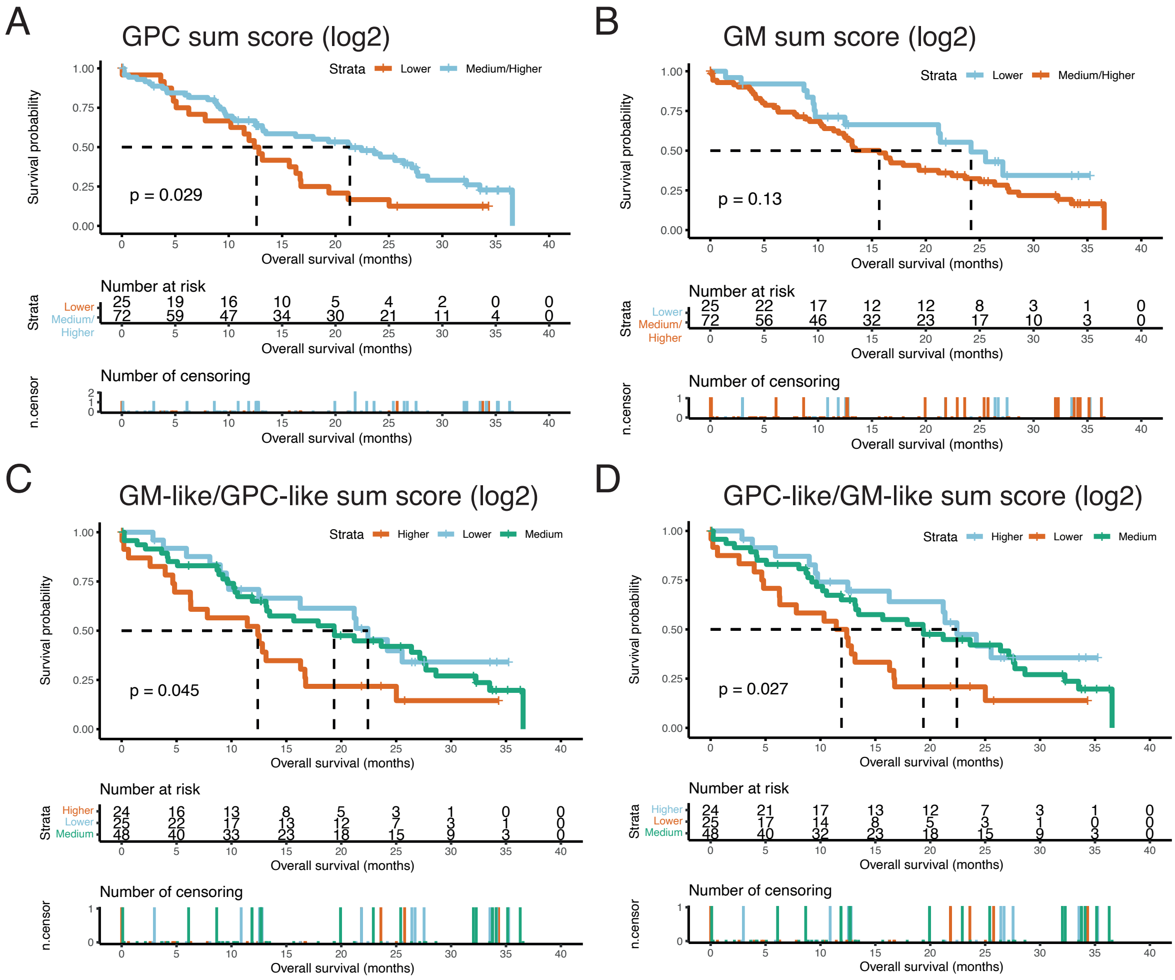


**Figure S12. Overall survival in GBM patients based on expression of the refined GSAPS, Kaplan-Meier (KM) curves, CPTAC data. A.** KM curves showing survival differences in patients categorized based on log2 GPC-like protein sum score expression to group of low (< first quartile) and medium/high (> first quartile) scores; **B.** KM curves showing survival differences in patients categorized based on log2 GM-like protein sum score expression to group of low (< first quartile) and medium/high (> first quartile) scores; **C.** KM curves showing survival differences in patients categorized based on log2 GM-like/GPC-like ratio to group of low (≤ first quartile), medium (> first and ≤ third quartile), and high expression (> third quartile); **D.** KM curves showing survival differences in patients categorized based on log2 GPC-like/GM-like ratio to group of low (≤ first quartile), medium (> first and ≤ third quartile), and high expression (> third quartile). The p values are based on log rank tests; the dashed lines present the median overall survival in the corresponding groups.

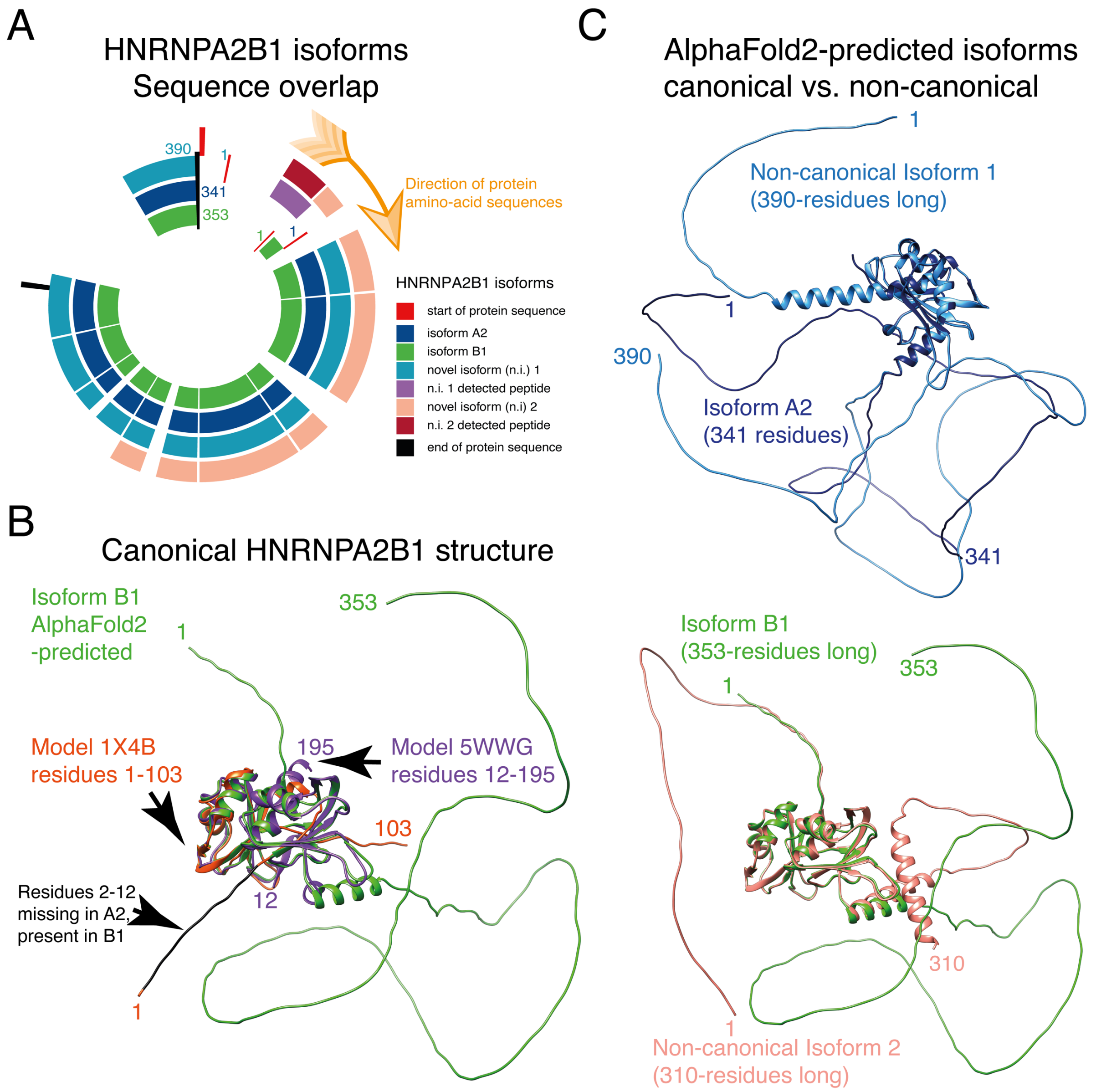


**Figure S13. Prediction of protein structure of canonical and non-canonical isoforms of HNRNPA2B1 with AlphaFold2. A.** Sequence overlap of HNRNPA2B1 isoforms; **B.** Canonical protein structure of isoform B1 predicted with AlphaFold2 (full-length sequence) overlapped with existing experimental models of HNRNP2B1 sequences – model 1X4B representing amino-acid residues 1 to 103 obtained with nuclear magnetic resonance and model 5WWG representing residues 12 to 195; **C.** AlphaFold2-predicted protein structure of HNRNPA2B1 isoforms. Up: isoform A2 overlapped with the protein structure of the novel isoform 1 – notice the alpha helix predicted in the 5'-UTR region of the novel isoform. Down: isoform B1 overlapped with the protein structure of the novel isoform 2.
